# Temperature and nutrients alter the relative importance of stochastic and deterministic processes in the coastal macroinvertebrates biodiversity assembly on long‐time scales

**DOI:** 10.1002/ece3.11062

**Published:** 2024-02-22

**Authors:** Xuhao Wan, Yuan Fang, Yueming Jiang, Xueqiang Lu, Lin Zhu, Jianfeng Feng

**Affiliations:** ^1^ College of Environmental Science and Engineering Nankai University Tianjin China

**Keywords:** assembly process, co‐occurrence pattern, marine macroinvertebrates diversity, nutrients, temperature

## Abstract

Macroinvertebrates play a vital role in coastal ecosystems and are an important indicator of ecosystem quality. Both anthropogenic activity and environmental changes may lead to significant changes in the marine macroinvertebrate community. However, the assembly process of benthic biodiversity and its mechanism driven by environmental factors at large scales remains unclear. Here, using the benthic field survey data of 15 years at large spatial and temporal scales from the Yellow Sea Large Marine Ecosystem, we investigated the relative importance of environmental selection, dispersal processes, random‐deterministic processes of macroinvertebrates community diversity assembly, and the responses of this relative importance driven by temperature and nutrients. Results showed that the macroinvertebrates community diversity is mainly affected by dispersal. Nitrogen and phosphorus are the most important negative factors among environmental variables, while geographical distance is the main limiting factor of β diversity. Within the range of 0.35–0.70 mg/L of nutrients, increasing nutrient concentration can significantly facilitate the contribution of the decay effect to β diversity. Within the temperature range studied (15.0–18.0°C), both warming and cooling can lead to a greater tendency for species diversity assembly processes to be dominated by deterministic processes. The analysis contributes to a better understanding of the assembly process of the diversity of coastal marine macroinvertebrates communities and how they adapt to global biogeochemical processes.

## Graphical abstract

The assembly mechanism of macroinvertebrates diversity in offshore waters is dominated by the dispersal process and random model, and this trend is maintained on a long‐time scale.

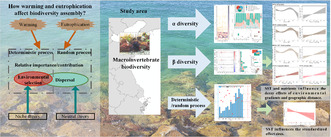

## 1 INTRODUCTION

Exploring the mechanisms of biodiversity formation and maintenance has been a central topic in community ecology research. The community assemblage formed by the diffusion of multiple potential interacting species is called the metacommunity. This concept provides ideas and methods for understanding the impact mechanism of spatial processes on community formation and has gradually become a focus in the ecological field (Corte et al., 2018; Leibold et al., 2004; Meynard et al., 2013; Paine, 2006; Rodil et al., 2017). The two established theories of community construction are niche‐based theory (Hutchinson, 1959; Silvertown, 2004) and neutral‐based theory (Bell, 2000; Hubbell, 2006; Sloan et al., 2006), which are reflections of deterministic and stochastic processes (Chen et al., 2019) in communities, respectively. Existing research shows that deterministic and stochastic processes are not absolute opposites, but rather complement each other and play a role together (Bahram et al., 2016; Roguet et al., 2015). In the course of different hypotheses and theoretical developments, after combining many frameworks and drawing on population genetics, the theory of community ecology was proposed by Vellend (2016). The construction of ecological communities was integrated into four higher‐order processes, including ecological drift (stochastic process), selection (deterministic process), dispersal (both involved), and speciation (both involved) (HilleRisLambers et al., 2012). Recent research has shown that assemblage communities are not described by four completely discrete models, but should be characterized as a continuum of different processes that differ in their relative importance (Logue et al., 2011; Ning et al., 2020; Zhang et al., 2021). However, intending to understand the process of community structure, quantifying such ecological processes, and dissecting the mechanisms of diversity assembly is a long‐standing and challenging problem (Zhou & Ning, 2017). The key point in solving this problem is to determine the relative importance of different ecological processes (ecological drift, selection, dispersal, and speciation) for the assembly of community diversity (Chase, 2010; Evans et al., 2017).

As an important component of marine ecosystems, macroinvertebrate communities are subject to multiple stressors (Cartaxana et al., 2015; Kosari et al., 2021; Lu et al., 2021; Saxena et al., 2015). Ocean eutrophication due to widespread human activities in combination with climate warming are the two main drivers of biodiversity loss in recent decades, either directly or indirectly (Newbold et al., 2015; Wang et al., 2016). Marine ecosystems have a stronger, more direct, and detectable response than terrestrial ecosystem (Pinsky et al., 2019; Poloczanska et al., 2013; Sunday et al., 2012) due to, among other things, reduced dispersal in response to isothermal changes (Burrows et al., 2019; Lenoir et al., 2019) and the relatively limited thermal micro‐refuges in the ocean (Suggitt et al., 2018; Sunday et al., 2015). Temperature determines metabolic rates, and warming may substantially alter benthic biodiversity through effects on ecological dynamics (Guo et al., 2019), community structure (Guo et al., 2018), and functional gene composition (Xue et al., 2016) and activity (Metcalfe, 2017). On the contrary, human activities are dramatically perturbing biogeochemical cycles, affecting the availability of important elements such as nitrogen and phosphorus (Hutchins & Fu, 2017; Kitidis et al., 2017). Nitrogen and phosphorus loads are increasing due to fossil burning, agricultural activities, and others (Battye et al., 2017; Peñuelas et al., 2013). Changes in the concentration of nutrients can often mediate changes in benthic community biodiversity by affecting species interactions (Dodson et al., 2000) and even alter ecosystem structure and function (Ardón et al., 2021; Baggett et al., 2013; Guignard et al., 2017). Recent comprehensive analyses reveal that nitrogen (LeBauer & Treseder, 2008) and phosphorus limitation (Hou et al., 2020) among aquatic primary producers on a global scale. These phenomena are expected to affect benthic fauna through food webs and energy transfer (Nessel et al., 2021; Pöyry et al., 2017). The interaction of temperature and nutrients on various organisms has been demonstrated and thus influences benthic growth and communities in different ways (Endo et al., 2013; Gao et al., 2017; Lomas & Glibert, 1999; McElroy et al., 2015). The effects of stressors may differ between different environments. However, there are fewer studies dealing with different environmental drivers of assembly mechanisms or the effects of stressors on benthic communities.

Among the different driving patterns of diversity assembly, dispersal drivers are more complex, compared to other ecological processes, because macroinvertebrates do not attach permanently to substrates (Pilditch et al., 2015), and many macroinvertebrates with pelagic larval stages (such as *Mytilus edulis*) are capable of dispersing and settling to new sites through stochastic processes such as strong waves, currents, and tides (Josefson & Göke, 2013). Both food availability and temperature or salinity gradients could influence the timing of spawning, mortality, or settlement behavior of marine macroinvertebrates larvae, which could further alter larval dispersal behavior (James et al., 2019). In addition, the relative importance of stochastic processes can vary between different aquatic organisms, with a higher proportion of stochastic processes in fish than in algae versus macroinvertebrates. (Göthe et al., 2017; Zhou et al., 2020). Scale and geographic distance also affect the contribution of stochastic processes. Often, large scales are dominated by stochastic processes (Heino et al., 2010; Wu et al., 2014), while small scales are better for deterministic processes (Qu et al., 2018; Zhou et al., 2020). A growing number of studies demonstrate the relative importance of stochastic and deterministic processes in aquatic communities' assembly (Göthe et al., 2017; Qu et al., 2018; Wu et al., 2018), but there are still few studies describing the importance of climatic and trophic factors in shaping different processes (Benito et al., 2018; He, Chen, et al., 2020; He, Wu, et al., 2020). The lack of widespread implementation of the theory of metacommunities with different process mechanisms translated into practical adaptive management hinders the conservation and restoration of marine biodiversity and ecosystem functioning.

Marginal seas account for 7%–10% of the global ocean area, but they have 30% of the global primary productivity and up to 80% of the global organic carbon buried in sediments (Bauer et al., 2013; Fang et al., 2018). As the typical marginal seas of the western Pacific Ocean, the Yellow Sea Large Marine Ecosystem (YSLME) plays an important role in protecting biodiversity, regulating climate, and other convenient and great economic values (Hu & Zhang, 2016). Anthropogenic activity and environmental changes are causing stress on the YSLME, and YSLME has gradually become a nutrient reservoirs after receiving large amounts of pollution input from land‐based sources (Zhao et al., 2018). Several studies have demonstrated the development process and importance of biodiversity in the YSLME in the past decades (Jin, 2004; Witkowski et al., 2016; Yu et al., 2018; Zhou et al., 2012). However, these studies often focused on a single sea area or small bays (He, Chen, et al., 2020; He, Wu, et al., 2020; Li et al., 2017), and the ecological processes have been inferred from only a few voyages, due to the lack of data over large‐scale and long‐time series.

Here, using the 15‐year marine macroinvertebrate communities data in the YSLME, we aim to: (1) identify the spatial and temporal dynamics of the relative contributions of environmental selection and dispersal limitation to α and β diversity in marine macroinvertebrate communities at large spatial and temporal scales; (2) reveal community random‐deterministic and co‐occurrence patterns; and (3) explore the mechanisms of temperature and nutrients mediation of macroinvertebrate community diversity assembly process. By exploring the mechanisms of macroinvertebrate community aggregation in the YSLME, the present study will contribute to an in‐depth understanding of the interactions between the environment and organisms, and provide new insights into the regulation of biological assembly processes by temperature and nutrients.

## 2 MATERIALS AND METHODS

### 2.1 Data

#### 2.1.1 Study area and nutrients data

**FIGURE 1:**
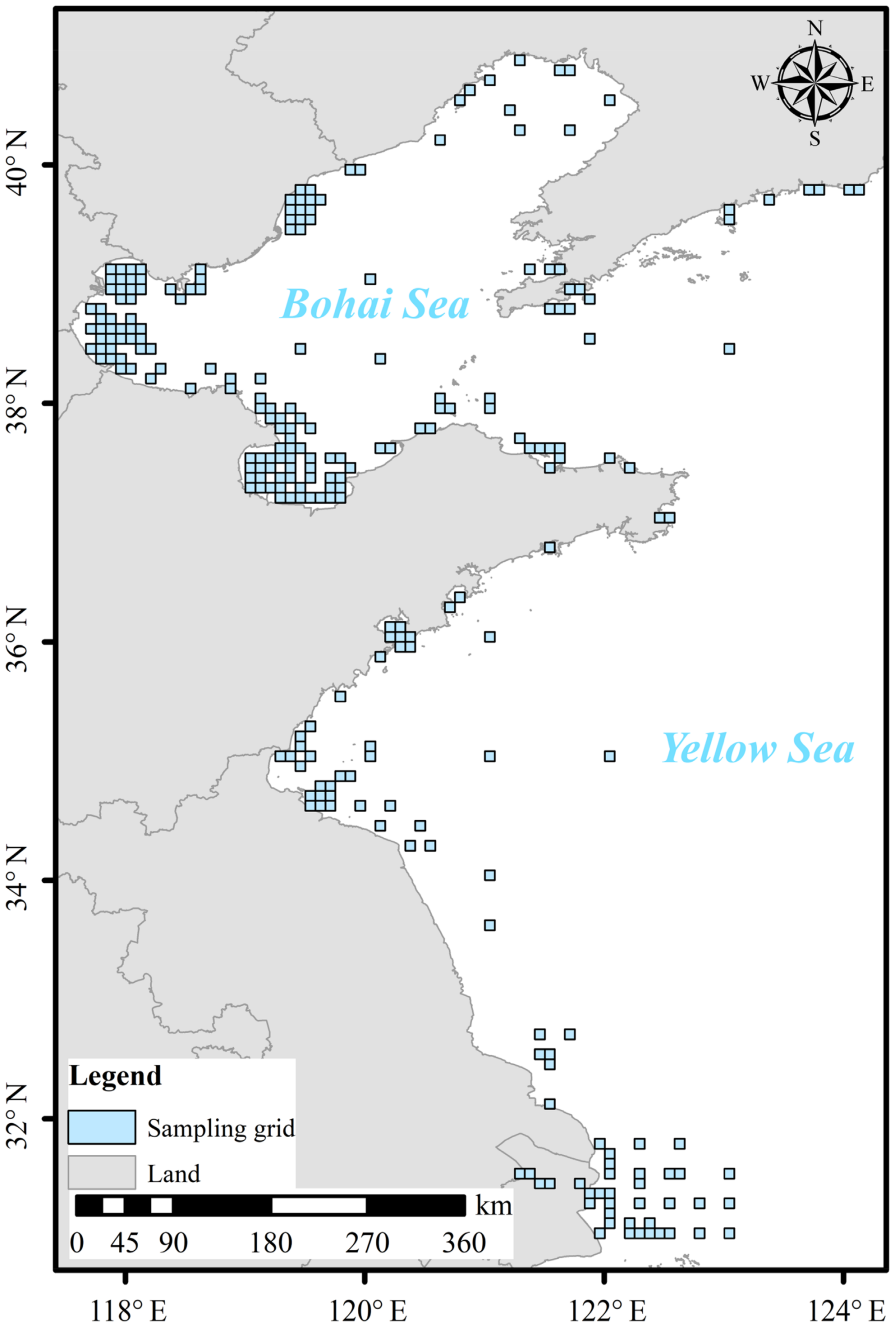
Distribution of grid points for statistical analysis (A total of 255 sites and over 1.2 × 10^4^ sample pairs per year were used).

The study area is in the coastal waters of the YSLME (Figure 1), which consist of the Bohai Sea (37°07′ N–41°0′ N, 117°35′ E–121°10′ E) and part of the Yellow Sea (35°00′ N–45°00′ N, 120°00′ E–123°00′ E), with water depths of approximately 0–50 m. Therefore, depths are shallow, and the water column is well‐mixed, so there was no thermocline. The area is located in the western part of the North Pacific Ocean, surrounded by land on three sides, and is a typical semi‐enclosed shelf‐edge sea with a total area of 4.6 × 10^5^ km^2^ (Zheng & Zhai, 2021). The hydrographic conditions are complex. The Yellow Sea warm current and the Yellow Sea coastal current are weak in summer and strong in winter. The coastal tides consist of regular and irregular half‐day tides, and the coastal water temperature varies greatly in all respects due to latitude and influencing factors, and all have obvious monsoon characteristics (Hu et al., 2012; Qiao et al., 2017).

The annual mean sea surface temperature (SST) data for the YSLME were obtained using the National Aeronautics and Space Administration (NASA) MODIS AQUA's SST product (MODSA‐AN4D9) with a spatial resolution of 0.25° × 0.25° and the time covered was from 2004 to 2018. The nutrient variable data of each site every year, including ammonium nitrogen (NH_3_–N/mg/L), nitrite nitrogen (NO_2_–N/mg/L), nitrate nitrogen (NO_3_–N/mg/L) and phosphate ($PO_{4}^{3+}$/mg/L) were obtained from historical survey data from the National Data Centre for Marine Science (NDCMS) for the entire YSLME, covering the period from 2004 to 2018. NH_3_–N, NO_2_–N, NO_3_–N, and $PO_{4}^{3+}$ were sampled from the mixed water column and analyzed according to the Chinese national standard methods for marine monitoring (GB 17378.4‐2007, 17378.6‐2007). The detection methods are indophenol blue, chromium‐copper reduction, diazotization‐coupling method, and phosphorus‐molybdenum blue method, respectively.

#### 2.1.2 Macroinvertebrate species data

Both macroinvertebrate Shannon‐Wiener diversity data and species presence‐absence data were derived from historical survey data from the National Data Centre for Marine Science (NDCMS) in the offshore waters of YSLME, from 2004 to 2018 (see Supplementary Data), and projected onto a 5′ × 5′ resolution equal‐area grid to generate a map of species diversity in the offshore waters of YSLME of China. The benthic survey methodology follows the national “Specifications for the oceanographic survey ‐ Part 6: Marine biological survey” (GB/T 12763.6‐2007). The benthic organisms were sampled using a 0.05 m^2^ grab mud collector, while the mud samples were later panned using a 0.5 mm sieve to sieve out the benthic samples. The obtained macroinvertebrates were fixed in a 5% neutral formaldehyde solution, and species identification was performed indoors. Small benthic organisms were species‐identified under a light microscope, and the relevant sequences from the macroinvertebrates survey were summarized by taxon.

As the distribution of survey stations varied between different years, to ensure consistency of spatial variables calculated by the Principal Coordinate of Neighbor Matrices (PCNM) and avoid bias in the study results under different spatial structures, the sites sampled more frequently than 30% of the time during the 15 years were retained, that is, the macroinvertebrates at the sample site were collected on average every 3 years. The final distribution of sampling sites is shown in Figure 1, with 255 sites and an average database of over 1.2 × 10^4^ sample pairs each year. To supply the α diversity data at missing sites in some years, the data from uncollected sites were extrapolated based on actual data, besides nutrients data, including dissolved oxygen (DO) and chlorophyll (Chl) data (correlation analysis only to impute diversity, collected from NDCMS). The minimal‐adequate model for each year was obtained based on a generalized linear model (GLM) (Table S1) for the selection of variables most relevant to diversity. These variables were brought into a co‐kriging interpolation to obtain the final results. All macroinvertebrates and environmental data were compiled on the same grid for the same years (Tittensor et al., 2010).

### 2.2 Data analysis

#### 2.2.1 Research framework

**FIGURE 2:**
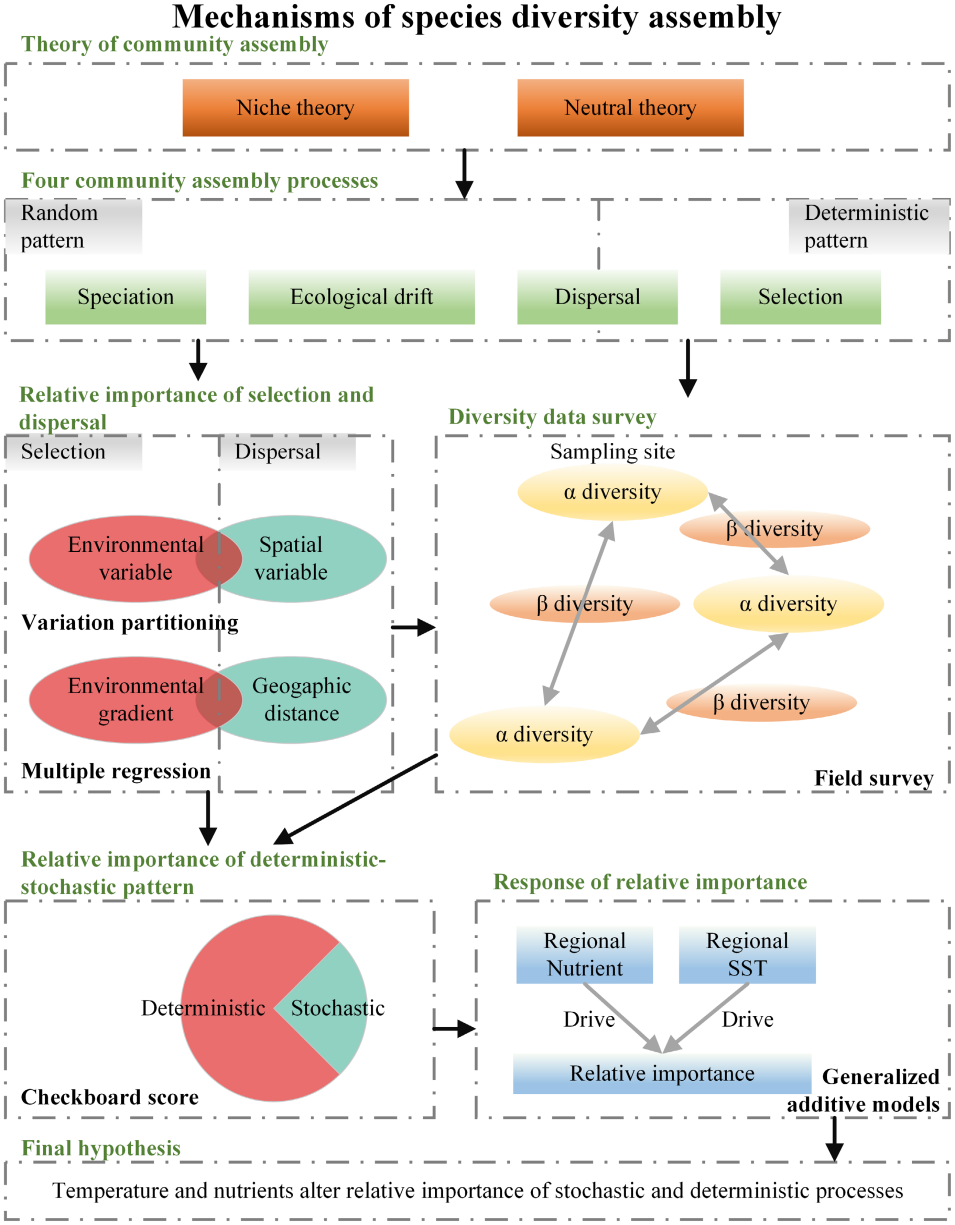
Research framework and flow diagram.

Based on the assembly mechanism and existing databases of species diversity, this study investigated the relative importance of environmental variables and spatial variables on the local scale for α diversity, to characterize the dominant role of selection and dispersal. Similarly, the decay effects of β diversity on environmental gradients and geographic distance were analyzed on the between‐site scale, that is, β diversity decreases with the distance decay, and similarity increases with the distance decreasing. This also indicates the relative importance of selection and dispersal. At the regional scale, the dominant role of stochastic‐deterministic patterns among species was investigated by comparing the significance of differences between empirical distributions and null‐model distributions based on randomization algorithms. Finally, a non‐linear regression fit was used to reveal trends in the influence of regional temperature and nutrient variation on the relative importance of each process in the three levels (α, β, and stochastic‐deterministic patterns). To explore the temporal dynamics of the assembly process, the data was processed and analyzed in the same way for each year (Figure 2).

#### 2.2.2 α diversity

A simple Pearson correlation analysis was first conducted to determine trends in variables and α diversity. To go further and compare the relative importance of SST, nutrients, and spatial variables on macroinvertebrate α diversity, the spatial structure of the study area needs to be quantified, and the approach typically adopted is to model the paired physical distance between sites to infer dispersal processes and understand lateral and vertical connectivity (Altermatt, 2013; Göthe et al., 2017; Heino et al., 2017). The spatial vectors were calculated with the Principal Coordinate of Neighbor Matrices (PCNM) method referring to the approach of Borcard et al., 2004, as shown in Figure S1. The calculation of the PCNM was carried out in the “SoDA” and “adespatial” packages in R 4.1.3 (Dray et al., 2006). SST, nutrient variables, and calculated spatial variables were used to diagnose covariance and forward selection for α diversity, with the criterion for selection of statistical significance at the 0.05 level to filter out significant key variables from environmental factors (NH_3_–N, NO_2_–N, NO_3_–N, $PO_{4}^{3+}$ and SST) and spatial variables. The relative influence of different types of environmental variable (SST and nutrients) selection and dispersal on the process of community α diversity construction was determined by using variation partitioning in multiple partial regression, with a transformation of log(*x* + 1) all biological data and environmental data (Borcard et al., 1992; Peres‐Neto et al., 2006). These steps were carried out in CANOCO 5.0. A Wilcoxon signed‐rank test for paired samples was conducted in IBM SPSS Statistics 26.0 to determine the dominant assembly process within the time series.

#### 2.2.3 β diversity

Differences in species composition between communities arise from two processes: species turnover or replacement and species loss or gain (Harrison et al., 1992; Willig et al., 2003). Turnover represents species replacement between communities, while species loss or gain can lead to differences in species richness between communities. When differences in species richness are ordered along some gradients, the community with fewer species will be a subset of the community with more species, thus showing a nestedness pattern (Lennon et al., 2001; Patterson & Atmar, 1986; Staniczenko et al., 2013). For β diversity, the Baselga approach (BAS) was used to calculate and partition β diversity, where the Sørensen pairwise dissimilarity index (β_sor_) was calculated with spatial turnover (β_sim_) and nestedness components (β_nes_) decomposed (Baselga, 2010; Lennon et al., 2001). Table S2 shows the diversity index equations, which were conducted in the package “betapart” of R 4.1.3.

To explore the decay (or increasing) effects of β diversity across gradients and distances, a matrix of geographic distances was calculated using the “geosphere” package in R, based on the GPS coordinates of each grid. The matrices of composite environmental variables were obtained by calculating Euclidean distances for SST and nutrient variables from the “vegan” package. To determine the relative importance of geographic, local SST, and nutrient factors in the construction of marine benthic communities, multiple regression analyses were conducted using the method of multiple regression on dissimilarity matrices (MRM), which features the calculation of similarities or distances between samples based on multivariate data and the construction of similarity matrices to replace the original multivariate data matrix. By performing regression analysis on multiple matrices to explain the overall effect of one set of variables on another, and using non‐parametric methods of permutation testing to determine the significance of regression coefficients, it facilitates the resolution of high‐dimensional biological data. Based on the regression coefficients and significance, the drivers that have the greatest effect on the response variables can be screened out. (Legendre et al., 1994; Lichstein, 2007). Specific environmental variables with strong covariance were screened before the application of MRM. The results showed that variables with high correlations (Spearman correlation coefficient > 0.8) were removed from the MRM analysis (only the correlation of NO_2_–N and NO_3_–N > 0.8 in 2005, so NO_3_–N was retained). Still, all other variables in the model were retained, based on which backward selection was performed to reduce spurious relationships between variables. The matrix data were then standardized for comparability among regression coefficients using the “MuMIn” package before the final MRM was performed using the “ecodist” package (Goslee & Urban, 2007; Wang et al., 2017). To account for zero similarity values, the community β diversity, as well as distance matrices, were ln(*x* + 1) transformed (Green et al., 2004; Talbot et al., 2014). Wilcoxon signed‐rank test for paired samples was also conducted to determine the dominant decay effects within the time series.

#### 2.2.4 Random and co‐occurrence patterns

The species co‐occurrence pattern was tested by examining the deviation of the empirical distribution from the mean of the null model, called the checkboard score (C‐score) (Stone & Roberts, 1990). The empirical distribution quantifies species co‐occurrence in pairs, that is, using the β_sor_. The null model was simulated by redistributing species coexistence through a randomization algorithm, and this study obtained the C‐score null distribution based on 30,000 random permutation simulations. The deviations are normalized to obtain the standardized effect size (SES) for comparison between assembly communities, with higher or lower SES values than expected for the null model indicating a predominantly deterministic process on the assemblage, and the higher the absolute value, the higher the degree of determinism(Crump et al., 2009; Swenson, 2014). SES values above or below the expected zero value are interpreted as over‐ or under‐diversification, respectively, and the magnitude of the SES is interpreted as the strength of the influence of the deterministic process on the community's diversity. In contrast, SES close to zero indicates no significant differences and a predominantly random pattern on assemblage. All operations were performed in the “EcoSimR” package (Mo et al., 2021).

#### 2.2.5 Influence of temperature and nutrients on assembly patterns

The Generalized Additive Models (GAMs) were used to establish the expected non‐linear relationships between the relative importance of the processes in the benthic diversity assembly and temperature or nutrients. The GAMs summarized a series of smoothing functions on the mean and variance of temperature and, therefore, captured the non‐linear relationships (Mccaffrey, 1992). Three models were developed for each type of process, including two univariate models: one for the relationship between the average SST and the process, and one for the relationship between the average nutrients concentrations and the process. The other was a full model, including the average SST and nutrient concentration. As the relative importance values of the different processes ranged from 0 to 1, a quasibinomial distribution was used to fit the model for the environmental selection and dispersal processes. Models for stochastic versus deterministic processes were fitted with a Gaussian distribution. Based on generalized cross‐validation (GCV), with each explanatory variable being consistent across models, the underlying dimension of the smoother was set to 4 for average SST and 3 for average nutrient concentration. Only univariate models with statistical significance, and full models with both explanatory variables statistically significant, are shown in the main figure. The GAMs were implemented by the “mgcv” package in R 4.1.3. (Li, Su, et al., 2019; Li, Xu, et al., 2019; Saw et al., 2017).

## 3 RESULTS

### 3.1 Mediation of α diversity assembly pattern

#### 3.1.1 Spatial–temporal dynamics of Shannon‐Wiener diversity

**FIGURE 3:**
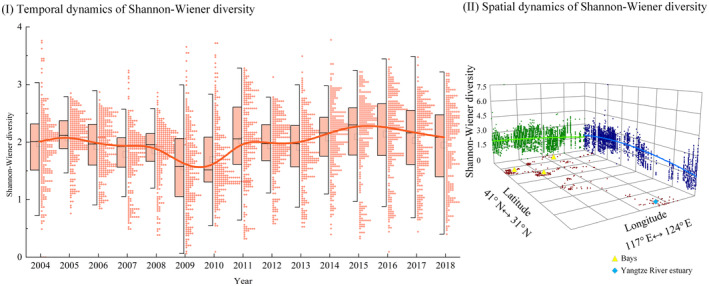
Temporal (I) and spatial (II) distribution of Shannon‐Wiener diversity. S and N represent the north–south trend, respectively. E and W represent the east–west trend, respectively. The green and blue points represent East–West and North–South projections, respectively. The green and blue lines represent East–West and North–South trend fits, respectively.

The spatial and temporal dynamics of Shannon‐Wiener diversity are shown in Figure 3. The Shannon‐Wiener diversity values for each year were mainly concentrated between 1 and 3 (Figure 3I), which indicates that the pollution status of marine ecosystems ranges from mild to moderate pollution based on the evaluation criteria (Qiu et al., 2018). Diversity underwent a decline in 2010, after which it seemed to peak in 2015–2016 (Figure 3I). Combining the historical survey data, the spatial distribution of diversity was obtained using co‐kriging interpolation. Diversity decreased somewhat from north to south. High diversity was mainly enriched in the three bays of the Bohai Sea, with the least macroinvertebrates at the Yangtze River estuary (Figure 3II).

#### 3.1.2 Relative contribution of environmental selection and spatial variables

**FIGURE 4:**
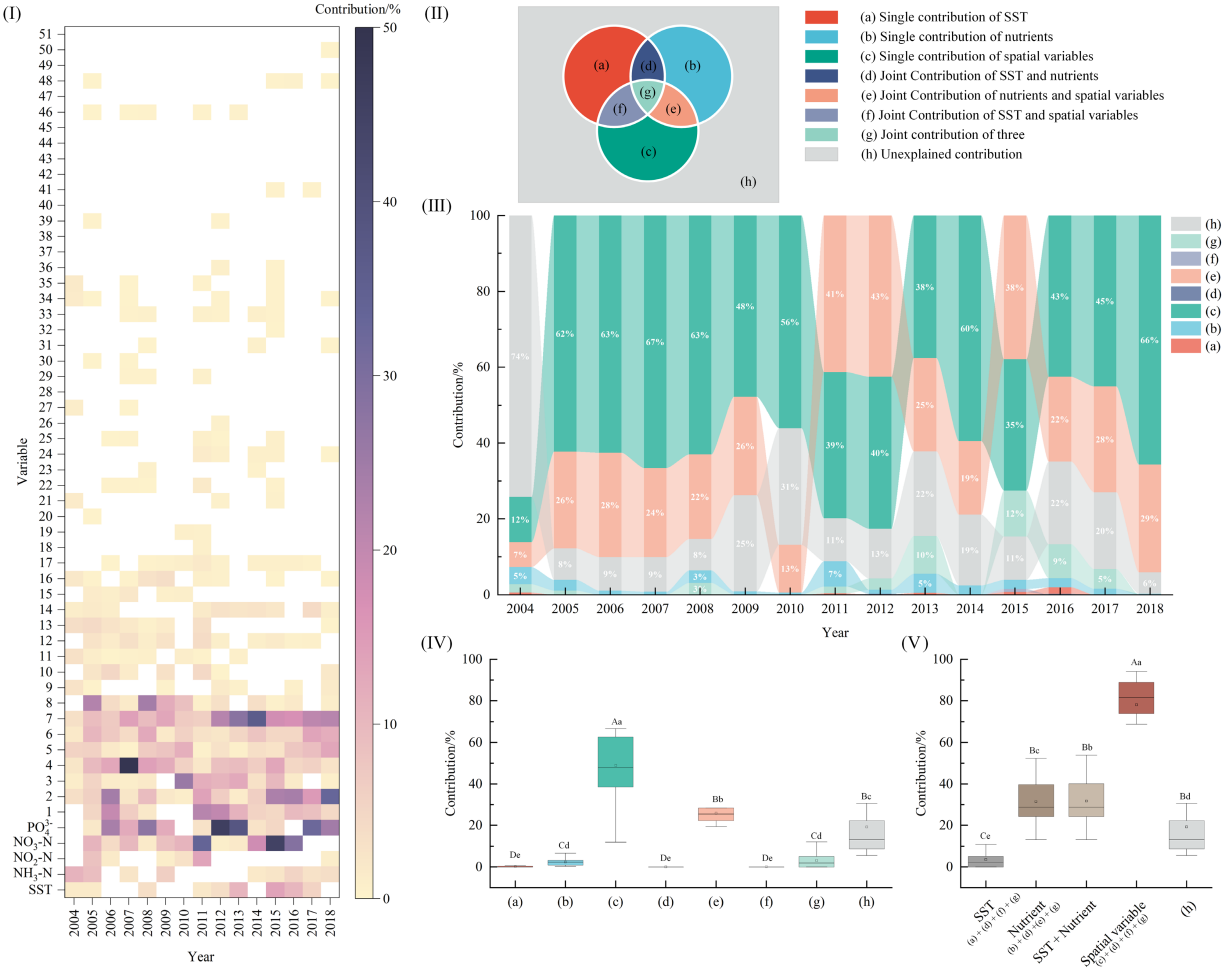
Relative contribution of SST, nutrients selection, and spatial variables to Shannon‐Wiener diversity (I) Univariate conditional effects on diversity (The fifty‐first spatial variable was numbered 1–51. Only significant contributions (*p* < .05) are shown in the figure, with blank sections indicating non‐significant effects). (II) Schematic diagram and legend of variance partitioning. (III) Relative contributions of different variables. (IV) Differences test results between individual contributions and joint contributions based on the Wilcoxon signed‐rank test of pair samples (Different upper‐ and lower‐case letters indicating significant differences at the 0.01 and 0.05 levels, respectively). (V) Differences test results between the three components based on the Wilcoxon signed‐rank test of pair samples (Different upper‐ and lower‐case letters indicating significant differences at the 0.01 and 0.05 levels, respectively).

Univariate Pearson correlation analysis shows that Shannon‐Wiener diversity was significantly negatively correlated with NO_3_–N and $PO_{4}^{3+}$ (*p* < .05). In most cases, diversity also had a significant negative correlation with SST. The effect of NH_3_–N NO_2_‐N on diversity changed over the years (Figure S2). After PCNM 51 spatial variables were obtained, covariance diagnosis and forward selection were carried out for them and nutritional variables, respectively, to obtain the final variables to be included in the model calculations, as shown in Table S3. The conditional effects of each univariate are shown in Figure 4I, with higher contributions to diversity from NO_3_–N (0–45.1%) and $PO_{4}^{3+}$ (0–46.0%). The higher contributions of spatial variables were concentrated in the first eight spatial variables (≤49.4%), with weak contributions from subsequent spatial variables (≤5.0%). The contribution of SST ranged from 0 to 13.7% only (Figure 4I). The results of the variation partitioning show that SST, nutrients, and spatial variables collectively explained at least 25.2% and up to 94.4% of the variation in diversity across years, with the smallest variance explained in 2004. In addition, each year had some degree of variation not explained by nutrients and spatial variables (5.6%–74.8%). The single contributions of spatial variables, joint contributions of spatial and nutrient variables, and unexplained contributions accounted for the top of the species variance each year. In contrast, the single contribution of SST, joint contribution of SST and nutrients, and joint contribution of SST and spatial variables explained the least diversity variation. Almost all of them explained less than 1%. In addition, no significant time trends existed (Figure 4III). Results of the difference analysis also show that the spatial variables alone led the way, followed by the joint spatial‐nutritional contribution, with the unexplained component being significantly higher than the other components (Figure 4IV). The results of the test of variance for the three components demonstrate that the contribution of spatial variables was the largest, suggesting that dispersal was dominant in the formation of α diversity (Figure 4V).

#### 3.1.3 Mediation mechanisms of environmental selection and spatial variable by temperature and nutrient shifts

Generalized additive models (GAMs) results for the contributions of annual average SST and nutrients to the contribution of environmental selection and spatial variables indicated that there was no single effect of annual mean SST on any of the different contributions (*p* > .05, Table S4, Figure S3). However, the full model considering both SST and nutrients indicated that the additive effects of both to the contribution of spatial variables were significantly non‐linear at the 0.1 and 0.001 levels (*p* < .01 and *p* < .001), respectively (edf > 1.000, Table S4), with a valley value in the effect of temperature at approximately 15.5°C, but not very significant (*p* = .0645, Table S4, Figure S3IV), so more data may be needed for validation. There was a sharp downward trend in the contribution of spatial variables at high nutrients, whereas there are no obvious fluctuations at low nutrients (Figure S4). A particular finding was the consistent trend in the prediction of spatial and total contributions from nutrients, confirming the result that spatial components dominated the assembly process of α diversity.

### 3.2 Mediation of β diversity assembly patterns

#### 3.2.1 Spatial–temporal dynamics of β diversity

**FIGURE 5:**
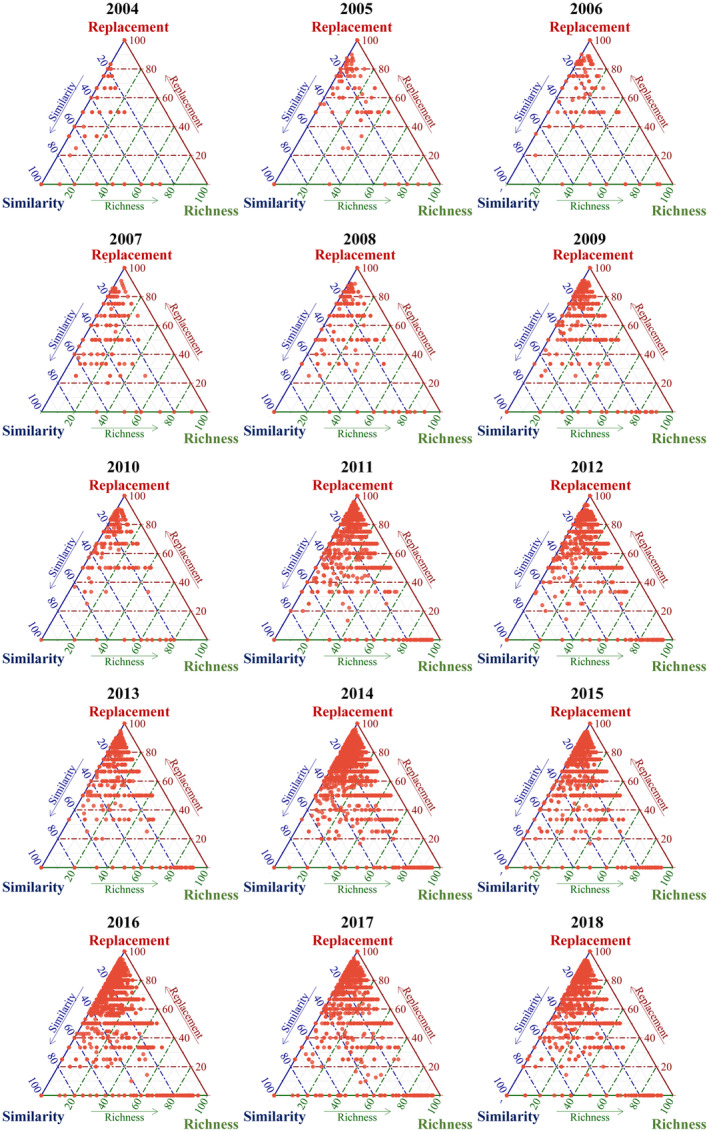
Distribution of similarity, spatial turnover components, and nestedness components for all sample pairs from 2004 to 2018.

**FIGURE 6:**
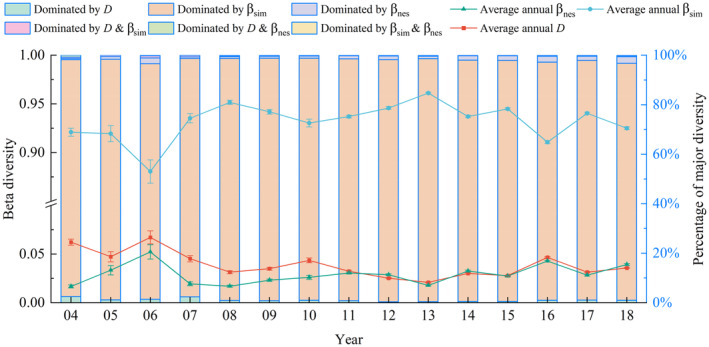
Annual average value of similarity, spatial turnover components, and nestedness components for all sample pairs with the proportion of sample pairs dominated by different components.

A large number of sample pairs of data concentrated in the upper triangular part of the ternary phase diagram suggests that most species diversity was dominated by β_sim_, with a low similarity and nested pattern between sample species (Figure 5). The same result is demonstrated by the dominance of the similarity, turnover, or nestedness components (Figure 6). The mean distribution for each year shows similar fluctuations and values for the similarity (*D*) and nested fractions (β_nes_), in contrast to the fluctuations for the spatial turnover fraction (β_sim_). The spatial turnover component was higher than the similarity and nested components in all years (Figure 6).

#### 3.2.2 The relative contribution of decay effects on environmental gradients and geographic distance

**FIGURE 7:**
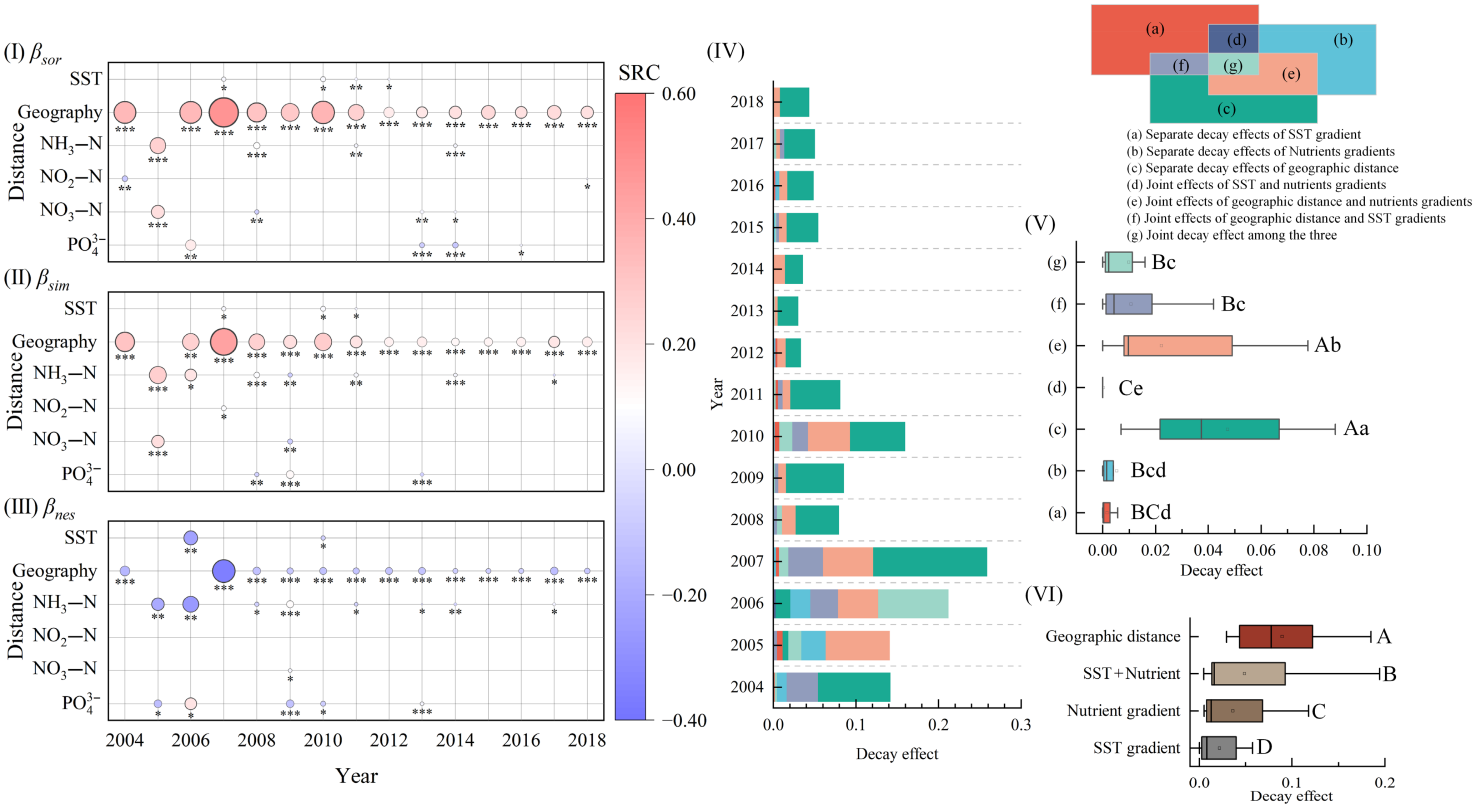
Relative contribution of the decay effects, β diversity on SST gradient, nutrient gradients, and geographic distance using the multiple regression analysis on matrices analysis (MRM). The partial standardized regression coefficients (SRC), including overall β diversity (I), spatial turnover components (II), and nested components (III), were calculated to express single distance decay effects (The partial regression coefficients and *p*‐values in the final model were reported from permutation test (nperm = 9999). The size of the circle represents the absolute value of the SRC. The larger the circle, the larger the absolute value of SRC. *, **, and *** denotes the significant correlation at 0.05, 0.01, and 0.001 levels, respectively.). Variation partitioning for β_sor_ was conducted based on MRM using Euclidean distances of multidimensional nutritional variables and geographic distance (IV). Differences test results between individual contributions, joint contributions (IV), and three components (V) based on Wilcoxon signed‐rank test of pair samples (Different upper‐ and lower‐case letters indicating significant differences at the 0.01 and 0.05 levels, respectively).

The distance decay relationship is one of the most common biogeographic distribution patterns, which means that community similarity decreases with the increase in geographical distance (Jiao et al., 2020). MRM was used to determine the relative decay effects of diversity across environmental gradients (Figure 7). The decay effect of β diversity for macroinvertebrates was largely explained by the MRM model at most environmental gradients (*p* < .001). Similarity (1–β_sor_) showed decay effects with geographic distance and nutrient gradients. The geographical distance was the most important variable explaining community β diversity (partially standardized regression coefficient, SRC = 0.17–0.48, *p* < .001). Except for 2005, the community similarity mainly showed a significant decline with the increase of NH_3_–N and NO_3_–N gradients (*p* < .001). The remaining nutrient gradients were ranked in importance after geographical distance (|SRC| < 0.17, *p* < .01, Figure 7I). The response of the spatial turnover fraction was similar to that of the overall β diversity, mainly because the majority of species diversity was dominated by the spatial turnover components (Figure 7II). In contrast, there was a gradual decrease in the nested fraction of species with increasing geographic distance and few environmental gradients, consistent with the finding that it fluctuated in the opposite direction to the spatial turnover fraction (Figure 7III). In addition, the effects on temperature gradients are small compared to geographical distance and nutrient gradients and are usually insignificant (Figure 7).

The results of the variance partitioning show that SST gradient, geographic distance, and variable nutrient gradients contributed differently to the decay effect of β diversity (Figure 7IV). The decay effect on pure geographic distance and the joint contribution of the decay effect on geographic distance and nutrient gradients were the two most dominant components of the effect (*p* < .01, Figure 7V). In most years, the separate contribution of geographic distance was also higher than the combined contribution of both (*p* < .05, Figure 7IV, V). The combined contribution of SST gradient and nutrient gradients was almost absent. However, there was some joint contribution among the three. Overall, the decay effect of β diversity in geographic distance dominated, especially in recent years, while the decay effect in SST gradients was the weakest (Figure 7IV–VI).

#### 3.2.3 Mediation mechanisms of decay effects on distance and gradients by temperature and nutrient shifts

**FIGURE 8:**
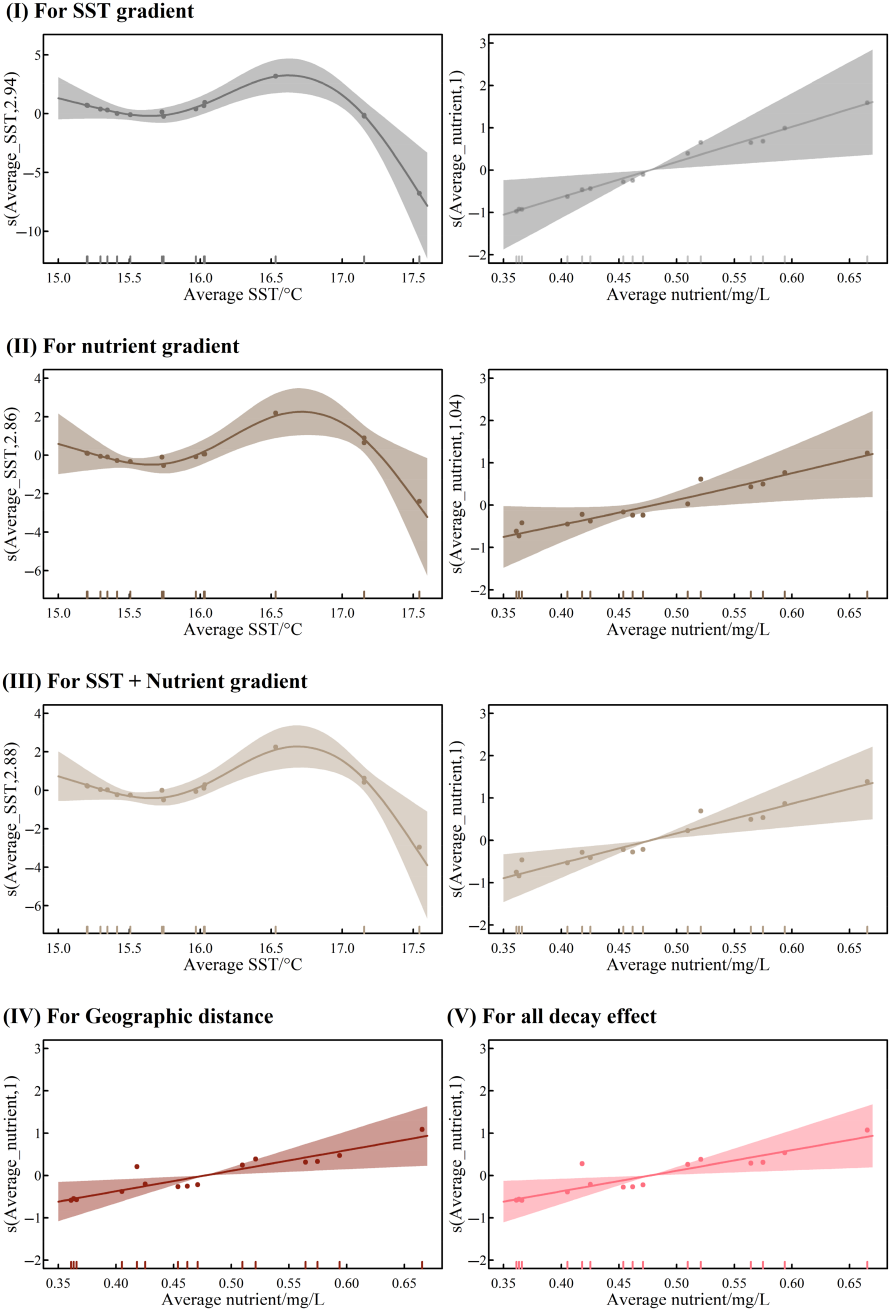
The impact of regional annual average SST and nutrients on the decay effects of SST gradient (I), nutrient gradient (II), SST + Nutrient gradient (III), geographic distance (IV), and all (V). (Average SST and nutrient refer to the regional average during the year. s() refers to the spline smoothing parameter, with the independent variables and the defined degrees of freedom in parentheses, respectively. The dots indicate the partial residuals.)

Average annual SST had a significant correlation and similar complex non‐linear trend in the decay effects of SST and nutrient gradients (edf > 1.000, *p* < .05, Table S5), and the effect decreases significantly after temperatures exceed 16.5°C (Figure 8I–III). In contrast, there was a significant linear increasing trend for all decay effects for nutrients (*p* < .05). Particularly, temperature and nutrients had additive effects in terms of the decay of environmental gradients. In contrast, decay effects on distance gradients, and all were only influenced by nutrients (Figure 8).

### 3.3 Mediation of species co‐occurrence patterns

#### 3.3.1 Temporal dynamics of species co‐occurrence patterns

**FIGURE 9:**
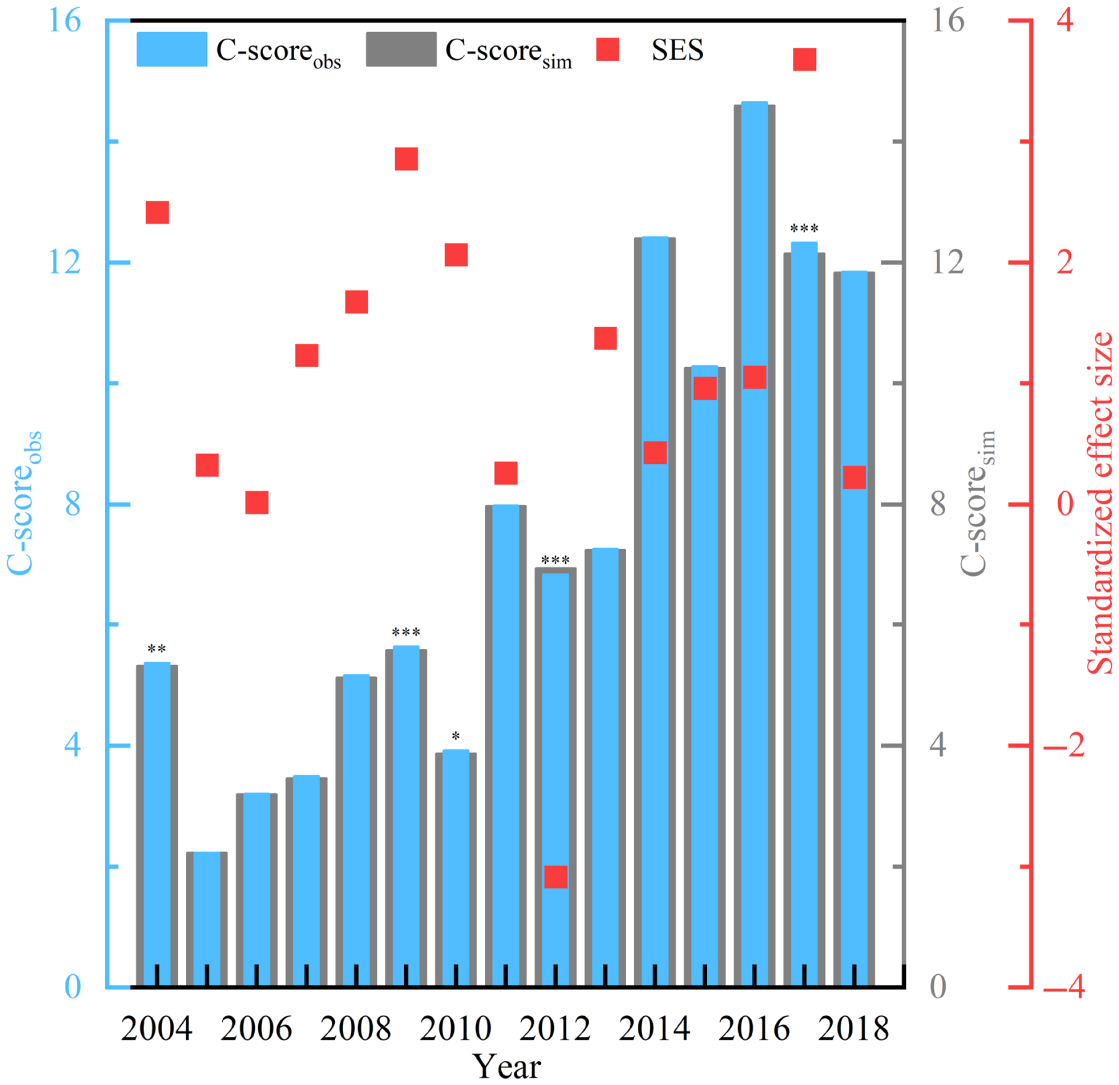
C‐score metric using the null model. (C‐score_obs_ indicate the observed C‐score based on the original species (0–1) matrix. C‐score_sim_ indicates the mean of the null distribution based on simulations obtained after 30,000 random permutations, a one‐tailed test of the observed and simulated values, and finally, a standardized effects size (SES). Observed C‐scores (C‐score_obs_) significantly higher or lower than simulated C‐scores (C‐score_sim_) values indicate deterministic co‐occurrence patterns (*, **, and ***). Standardized effect sizes (SES) < −2 and > 2 represent aggregation and segregation, respectively.)

The pattern of benthic community assembly varied over different years (Figure 9). In 2004, 2009, 2010, 2012, and 2017, the community assembly process was dominated by deterministic processes (*p* < .05), with species co‐occurring in an aggregation pattern (SES < −2) in 2012 and a segregation pattern (SES > 2) in 2004, 2009, 2010 and 2017, while the community assembly process within the other years was dominated by random processes (*p* > .05).

#### 3.3.2 Regulatory mechanisms of temperature shift

**FIGURE 10:**
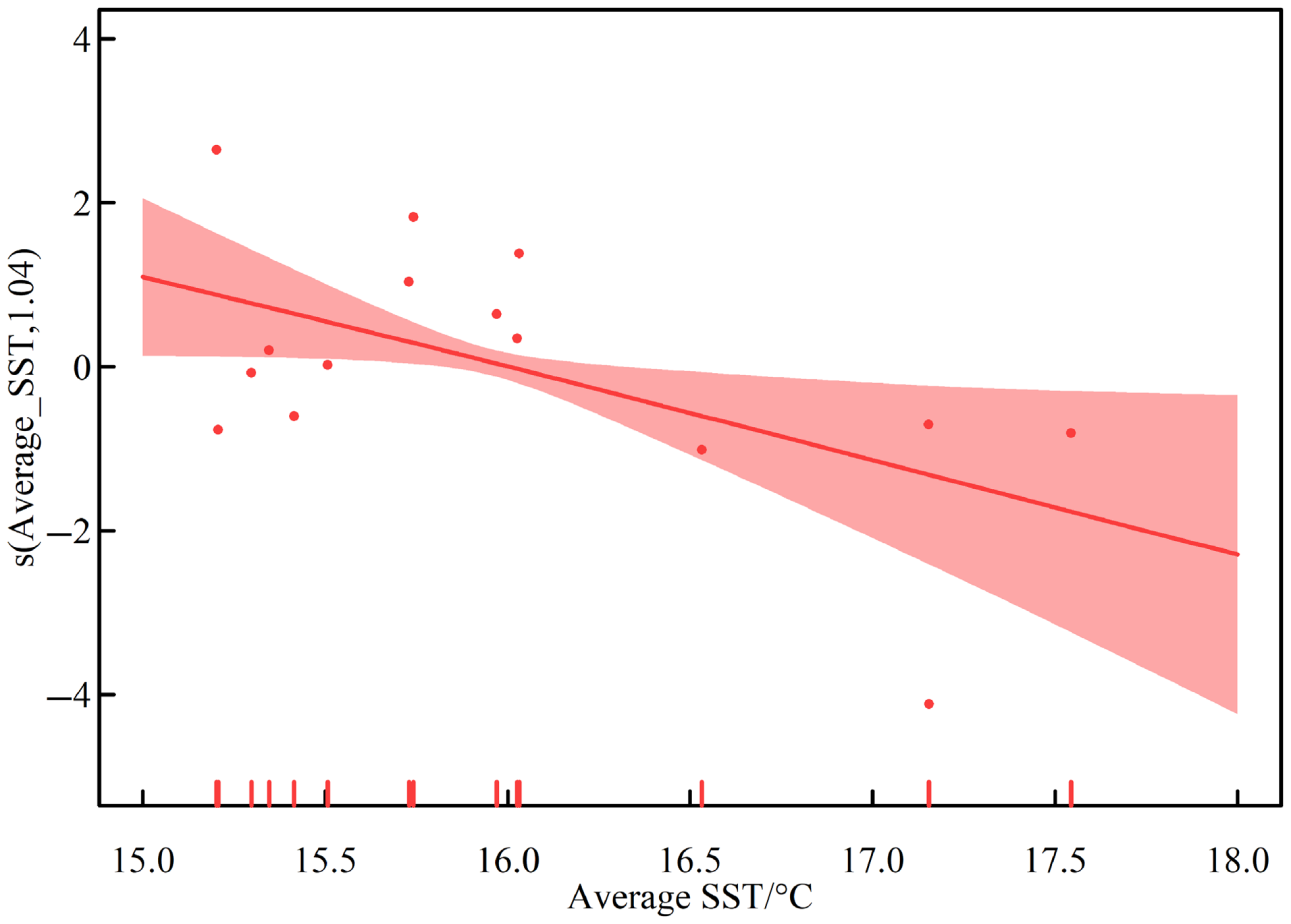
The impact of regional annual average SST on the standardized effect sizes. (Average SST refers to the regional average during the year. s() refers to the spline smoothing parameter, with the independent variables and the defined degrees of freedom in parentheses, respectively. The dots indicate the partial residuals.)

Changes in mean annual SST had a significant negative linear correlation with SES (edf = 1.000, *p* < .05), but variance did not have any effect on SES (Table S6, Figure 10). The lower the mean annual SST, the more species formation tended toward a segregated pattern, and the higher the mean annual SST, the more species formation tended toward an aggregation pattern. Both low and high temperatures increased the degree of certainty and decreased the degree of stochasticity in community formation processes (Figure 10). The sensitivity analysis shows that when the aggregation pattern is not considered without SES < −2, the trend of negative correlation between SST and SES is still present, but not statistically significant (Figure S7). Therefore, it could be confirmed that the effect of SST is not strong when only the segregation pattern with a stochastic process is considered. However, since the aggregation pattern was only observed in 2012 in this study, the data for that year are more sensitive. It could be predicted that the effect of temperature is more significant when more aggregated pattern data are found.

## 4 DISCUSSION

### 4.1 Diversity pattern of macroinvertebrate community in YSLME

The α diversity reflects the diversity of species within a sample site, while β diversity reflects the diversity of species between sample sites. Due to the differences in the structure and mathematical expressions of different indicators, different diversities always represent fundamental aspects of biomes (Socolar et al., 2016). Partitioning of β diversity is crucial to the understanding of core biogeographic issues, helping to clarify the processes involved in benthic community assembly and better understand the mechanisms by which patterns of diversity distribution are formed and stabilized (Ge et al., 2022; Podani & Schmera, 2011). Calculated nested values obtained after partitioning are not measured in an absolute sense. It reflects the fraction of dissimilarity due to the effects of nested patterns in the community. This value captures the non‐random process that promotes species order loss due to any factors (Baselga, 2010; Gaston & Blackburn, 2000). Conversely, spatial turnover represents the result of species being displaced by other species due to environmental filters, spatial isolation, or historical factors (Qian et al., 2004). The pattern of β diversity in the YSLME was dominated by spatial turnover of species, which appears to be a common phenomenon across a wide range of taxa in estuarine or marine ecosystems, such as aquatic plants (Bertuzzi et al., 2019), marine fishes (Liggins et al., 2015) and other benthic fauna (Alsaffar et al., 2019) in the ocean. This suggests that there is species turnover between sites, or that endemic species are present at one of the sites (Baselga, 2010). This may be due to community differentiation due to barriers between different sites. Therefore, in biological conservation, more attention should be paid to the conservation of different sites, a strategy that is also contrary to conservation based on nested models (Wright & Reeves, 1992). The lower diversity exhibited in the Yangtze River estuary region may be due to the rapid development of coastal cities in the area and a well‐developed port transport industry, hence the high land‐based input of nitrogen and phosphorus. The high concentrations of nitrogen and phosphorus harm benthic diversity, as shown by Pearson correlation results. The resulting benthic diversity was low.

### 4.2 The relative contribution of environment selection and dispersal to community diversity

Describing spatial patterns in ecosystems and identifying the processes and factors that influence them is a research objective in ecology. When spatial variables were included in the variation decomposition, our results show that spatial, nutrient, and temperature factors significantly explained variation in macroinvertebrate community diversity, although biodiversity was significantly correlated with different spatial and environmental variables over different years. More importantly, the purely spatial component was a better predictor of benthic communities, explaining the higher variation of diversity. This result confirms recent findings (Corte et al., 2018; Josefson, 2016; Rao et al., 2020; Rodil et al., 2017), and reinforces the relative importance of dispersal limitation in the assembly of community diversity in offshore ecosystems.

Geographical distance is one of the most commonly used proxies in dispersal limitation, meaning that closer communities are more likely to exchange individuals than communities farther apart (Moritz et al., 2013). For the β diversity, benthic communities strictly followed a distance‐decay relationship, with diversity increasing and similarity decreasing with increasing distance. There was also a significant partial relationship between benthic community diversity and nutrient gradients. After decoupling their effects on diversity variation, both geographic distance and nutrient gradient decay remained significant for benthic communities, but nutrient gradients had less explanatory power, suggesting that some of the spatial autocorrelations of species may arise from nutrient filtering. However, dispersal is likely to be influenced not only by geographical distance but also by hydrology and biological behavior (Moritz et al., 2013; Shanks, 2009). Dispersal is a key limiting factor in shaping community diversity in both α and β diversity, a pattern that is expected since the same species or functionally similar species share similar habitats within a short distance of each other (Cottenie, 2005; Soininen et al., 2007). Numerous studies have also found that since the 21st century, the size of echinoderms, crustaceans, and mollusks in the Bohai and Yellow Seas has been gradually replaced by small polychaetas and mollusks, with a tendency for communities to miniaturize (Jiang et al., 2023). The size‐plasticity hypothesis predicts that smaller organisms are more susceptible to dispersal constraints and are widespread across habitats due to their greater metabolic adaptations (Langenheder & Ragnarsson, 2007). In contrast, the size‐dispersal hypothesis suggests that fewer organisms are less affected by dispersal and, therefore, may disperse anywhere, reflecting only environmental influences (Farjalla et al., 2012). The results in this study appear to be more supportive of the size‐plasticity hypothesis, with dispersal more likely to affect miniaturized organisms. Changes in macroinvertebrate communities are driven by a combination of dispersal limitation and environmental filtering, supporting the general view that combinations of assemblage community models can simultaneously contribute to community change (Cottenie, 2005).

Another particular finding is that as the nutrient gradients and distance increase, the turnover component increase and nestedness decreased. This is because β_sim_ is not only a turnover measure but also an inverse measure of nestedness between samples, the proportion of poorer samples nested into richer samples (Carvalho et al., 2012). Similar trends have been found in related studies (Josefson, 2016). A common cause of geographical nesting may be due to habitat fragmentation. If fragmentation varies in size and isolation, nesting patterns can arise (Ulrich et al., 2009). The mechanism for it may lie in that smaller habitats selectively retain species with high abundance. If the habitat is isolated, it is difficult for these species to redispersal and recolonize. In the present study, the ocean at large scales can be considered as a fragmented habitat with widely varying areas and with different isolation due to distance and ocean topography.

In addition, the variability explained by spatial structure may also arise from some non‐measured variables (Corte et al., 2018). For example, oxygen in sediment has been confirmed to show strong spatial variation in offshore systems and can have a significant effect on macroinvertebrates (Quiñones‐Rivera et al., 2007; Riedel et al., 2012). Water depth was also found to influence macroinvertebrate communities, with deeper waters showing higher primary production near the bottom, thereby promoting macroinvertebrates (Xu et al., 2021). On the contrary, the large proportion of unexplained variation in both types of diversity could be attributed to species interactions (Godoy, 2019), unmeasured environmental variables (e.g., substrate complexity and sediment grain size are important unmeasured variables (Gething et al., 2020)) and stochastic processes (Gibert & Escarguel, 2019). Among other drivers, bathymetry, seasonal factors, and habitat fragmentation may also be factors affecting diversity. It has also been shown that water depth differences are more important in shaping the structure of benthic ciliate communities than geographic distance and environmental gradients (Li, Su, et al., 2019; Li, Xu, et al., 2019). Benthic foraminiferal diversity could also respond dependently to monsoon‐induced increases in carbon fluxes (Heinz & Hemleben, 2006). Habitat fragmentation is also a major threat to biodiversity, and the trophic shifts it induces could have transcendent impacts on food web structure and, stability, and ecosystem function (Layman et al., 2007). The results of the study only explored the relative importance between temperature, nutrients, and dispersal, but it did not mean that they are necessarily more important than other factors. The considerable unexplained variation supports this conclusion. It is therefore recommended to attempt to use gene sequencing for species identification and include additional levels of environmental variables in further studies (Alsaffar et al., 2020; Liu et al., 2022; Wing et al., 2018), to better understand the relative importance of processes governing the environmental selection and dispersal limitation.

### 4.3 Temperature and nutrients mediating assembly of macroinvertebrate communities

The nutrients mainly affect the relative balance of the spatial variable process in the formation of α diversity, and the contribution of environmental gradients and geographical distance to β diversity. A significant trend is that under the condition of high nutrients, the contribution of spatial variables decreases steeply. The higher nutrients could reduce nutrient limitations to organisms (Hillebrand et al., 2014; Vitousek & Howarth, 1991). Nutrient saturation leads to the transfer or deterioration of restrictive nutrients in the environment (Hillebrand et al., 2014; Nair, 2014). The phenomenon would aggravate the element imbalance and mismatch between consumers and resources through a top‐down or bottom‐up regulatory mechanism (Mitra & Flynn, 2005; Nijssen et al., 2017; Stevens et al., 2018). In addition, the rapid growth and reproduction of organisms could be restricted (Elser et al., 2009; Meunier et al., 2017). For example, the early developmental stages of some marine macroinvertebrates, as seemingly the most sensitive group, are subject to the toxic effects of nitrogen‐saturated deposition derivatives (Nijssen et al., 2017). A previous study has found that chronic nitrogen deposition in an ecosystem may lead to nitrogen saturation (Nair, 2014). Such a consequence is that species are mainly difficult to filter through the environment, reducing diversity, and are not affected by the spatial structure. The result of this trend fitting is mainly controlled by the high nutrient content in 2004. This year, the main source of high nutrition was the high concentration of NO_2_–N and NO_3_–N. After that, the concentrations of these two types of nutrients gradually decreased due to policy and ecological construction. On the contrary, all gradient contributions to β diversity increased with the increase in nutrient content. Based on the above nutrient restriction, the reproduction of specialized organisms, such as carnivorous invertebrates and N‐fixing organisms, is promoted (Vitousek & Howarth, 1991). Therefore, due to the difference in the sediment environment, different samples have different franchised organisms, which will increase β diversity. The potential mechanism of the phenomenon is possibly due to the restriction of environmental gradients and geographical distance.

The influence of warming on the assembly process of diversity seems more complex, not only affecting the spatial and environmental processes of α diversity but also changing the gradient effects of β diversity, and even controlling the pattern of species co‐occurrence. The possible explanation for the increased explanatory power of spatial structure under high temperature is that, for species that have passed environmental selection, the ambient temperature may have reached the maximum value of their thermal endurance coefficient, so the higher temperature will make them unfit for survival (Bates et al., 2014; Cheung et al., 2013; Deutsch et al., 2008). Elevated temperatures also affect the regulation of hemolymph osmotic and ionic concentrations in macroinvertebrates (Orr & Buchwalter, 2020). Some isopods shift from aerobic to anaerobic metabolism in response to warming (Modlin & Jayne, 1981). Experiments have also found that adult females are smaller than males at higher temperatures, which refers to that somatic cell growth and reproduction cancel each other out (McKie et al., 2004). In addition, climate change typically increases the metabolic rates of the organisms, increasing the likelihood that larvae would settle closer to the birth population (Figueiredo et al., 2014; Kendall et al., 2013). Although seasonal‐scale data were not available for this study, it is undeniable that seasonally anomalous temperatures can also cause adults to release larvae at suboptimal times (Tlusty et al., 2008). Prolonged exposure to higher temperatures reduces larval settlement (Randall & Szmant, 2009). Synergistic effects of temperature with other factors can also affect larvae. Higher temperatures can ameliorate the stress of low salinity on larvae (Przeslawski et al., 2015). Warming and acidification synergistically reduce the survival of a range of taxa in early life stages (Bashevkin et al., 2020; Harvey et al., 2013; Przeslawski et al., 2015). Interspecific competition, migration from different areas, and species dispersal all occur in search of limited thermal refuges. In the case of low temperature, the higher metabolism and species differentiation rate in an environment suitable for survival increases the species diversity (Clarke & Gaston, 2006; Rohde, 1992). Pearson correlation results also support this conclusion. With the increase in diversity, there will inevitably will be competition for resources among species, so it is important to spread to find more suitable habitats. On the contrary, warming breaks through the effect of weakening the environmental gradient. Perhaps it breaks through the threshold of heat tolerance, and a large number of species are not suitable for survival (Antão et al., 2020). Therefore, the species that survive at different sample points are not very different, and there is no significant decay effect on the environmental gradient.

Most studies have shown that co‐occurrence patterns differ at different spatial scales, but co‐occurrence patterns at other scales, such as temporal scales, have received little attention (Kohli et al., 2018; Zhu et al., 2021), particularly changes in temperature, which may be important for the survival of the marine benthos. Furthermore, community assemblages are more strongly influenced by deterministic processes in high or low‐temperature environments, possibly because deterministic processes tend to have a greater impact on habitat‐strict species. A new finding is that species co‐occurrence patterns tend to aggregate by warming while cooling causes species to tend toward stochastic processes and even form segregation patterns. There are several possible explanations. Based on the expectation of the limiting similarity principle, species that emerge in aggregations tend to have a lower degree of ecological niche overlap than species in segregation patterns (Macarthur & Levins, 1967). Therefore, warming increases metabolic rates and enzymatic activity (Mooshammer et al., 2017; Rohde, 1992), allowing most species to more easily pass‐through environmental filtering and reach suitable habitats, making co‐occurrence more likely than would be expected by chance (Ramalho et al., 2021). Then the result could lead to aggregation patterns, with potential process causes possibly being a similarity in habitat requirements or the presence of positive biological interactions (Gotelli & McCabe, 2002; Ovaskainen et al., 2010). On the contrary, environmental filtering constructs co‐occurrence patterns by promoting spatial isolation, and some functionally distinct species could aggregate. Patch dynamics can also provide a general description of marine systems. Asynchronous turnover of patches can create asynchronous landscapes in which species are far more likely to coexist than in homogenous environments. Explaining the life histories of species, exploring the existence of suitable and food‐rich habitats, and the ease with which species can reach them can therefore provide a better understanding of species co‐occurrence patterns (Corte et al., 2018). As temperatures drop, a large number of warm water macroinvertebrates' activity decreases (Li et al., 2013) and benthic diatoms become unsuitable for survival (Cheung et al., 2021), leading inevitably to a need for species to compete for warmer and more suitable habitats. The resulting migration or death of individuals and susceptibility to disturbance, dispersion, or other processes leads to a rather chaotic situation of randomization (Schamp et al., 2015) and even the formation of segregation patterns as temperatures continue to fall. Non‐random patterns of co‐occurrence may result from the competition as well as from shared environmental preferences (in this study, preferences were other variables at high temperatures and warmer environments at low temperatures), degrees of environmental heterogeneity, and differences in the geographical distribution of species (Bell, 2000; Heino et al., 2017). In addition, spatial temperature fluctuations due to local warming, enhance the explanatory power of dispersal limitation and decay patterns, possibly because the warming of parts of the ocean increases the dispersal of ocean currents and thus environmental heterogeneity, so that decay in either gradients or distance can be enhanced. Of course, no single mechanism will likely be sufficient to encapsulate the complexity that arises when multiple environmental, biological, and spatial processes come together to construct community diversity.

### 4.4 Limitation and expectation

The diversity trends displayed here are interpreted in the context of the ecosystem of China's offshore waters. On the one hand, although nutrient variables that are commonly thought to influence macroinvertebrate community structure were included in the study, many other variables were not considered. On the other hand, the effects of spatial structure were related to the location of sampling sites, which were selected as a result of screening for data availability. Such problems may lead to an underestimation of the explanatory power of the nutrient selection obtained from the research. Furthermore, studies of diversity should not be limited to a particular taxonomic level but should incorporate more levels of diversity, such as diversity of genes, traits, ecosystem services, and functions. Driving mechanisms should be explored from a more systematic and comprehensive perspective. On the contrary, the spatial distribution of the data and the time series are limited to adequate records, with a large proportion of the deep macroinvertebrate data far from the nearshore missing. Continued increased temperature and eutrophication in the future will deteriorate marine biodiversity, and their complex underlying mechanisms require further research. However, based on the complex and large‐scale region of the YSLME, the results of this study can provide a reference for research on the mechanisms of regional responses of benthic communities to global environmental effects.

## 5 CONCLUSIONS

The distribution pattern of coastal marine macroinvertebrates community diversity is controlled by a combination of selection by environmental factors (temperature and nutrients) and dispersal, with dispersal being the main process dominating the assembly patterns. Nitrate and phosphorus are the most significant negative factors for α diversity. And geographic distance is the main factor for limiting the β diversity. Compared to deterministic processes, stochastic processes are dominant in the assembly process, and the dominance of dispersal processes supports the result. Nutrient concentrations, at regional scales in the range of 0.35–0.7 mg/L, mediated a linear increase in the decay effect of beta diversity across environmental gradients and geographical distances. And temperature on a regional scale in the range of 15.0–18.0°C mediated a pattern of stochastic‐deterministic processes. It is shown that both warming and cooling over a range of temperatures allow deterministic processes to become more dominant, and to cause a greater tendency for species co‐occurrence patterns to aggregate and separate, respectively. Therefore, controlling coastal nutrient inputs and warming could facilitate macroinvertebrates community assembly toward greater diversity and promote sustainable ecosystem development.

## AUTHOR CONTRIBUTIONS

**Xuhao Wan:** Data curation (equal); formal analysis (equal); methodology (equal); writing – original draft (equal). **Yuan Fang:** Data curation (equal); investigation (equal). **Yueming Jiang:** Data curation (equal); investigation (equal). **Xueqiang Lu:** Conceptualization (equal); writing – review and editing (equal). **Lin Zhu:** Methodology (equal); supervision (equal); writing – review and editing (equal). **Jianfeng Feng:** Conceptualization (equal); funding acquisition (equal); project administration (equal); resources (equal); supervision (equal); writing – review and editing (equal).

## FUNDING INFORMATION

This research is supported with funds provided by the National Key Research and Development Program of China (2019YFE0122300, 2018YFC1406403).

## CONFLICT OF INTEREST STATEMENT

The authors declare no conflict of interest.

## Supporting information


Data S1.

## Data Availability

Space variable data uploaded as online.
