# Supplementary material for: Temperature and nutrients alter the relative importance of stochastic and deterministic processes in the coastal macroinvertebrates biodiversity assembly on long‐time scales
Source: Ecol Evol. 2024 Feb 22;14(2):e11062. doi: 10.1002/ece3.11062 (PMC10883258; doi:10.1002/ece3.11062)
Supplement: Supplementary file 1 — Data S1. [file ECE3-14-e11062-s001.doc]

**Supplementary Material and Appendices**

Table S1 Minimal-adequate GLM results of α diversity.

Table S2 Formula of β diversity and its partitioning.

Table S3 Final variables screened by covariance diagnosis and forward selection.

Table S4 Summary of GAM results for the model of the relationship between the contribution of SST, nutrient variables, spatial variables, all contribution and annual average SST and nutrient levels.

Table S5 Summary of GAM results for the model of the relationship between the decay effect of SST gradient, nutrient gradients, geographic distance, all and annual average SST and nutrient levels.

Table S6 Summary of GAM results for the model of the relationship between the standardized effect size (SES) and SST.

Fig. S1 Calculation of PCNM variables.

Fig. S2 The Pearson correlation between nutrients and Shannon-Wiener diversity every year.

Fig. S3 The effect of regional annual average SST on the relative contribution of processes in the construction of α diversity, using univariate GAMs analysis.

Fig. S4 The effect of regional annual average nutrient levels on the relative contribution of processes in the construction of α diversity, using univariate GAMs analysis.

Fig. S5 The effect of regional annual average SST on the relative decay effects of β diversity, using univariate GAMs analysis.

Fig. S6 The effect of regional annual average nutrient levels on the relative decay effects of β diversity, using univariate GAMs analysis.

Fig. S7 Impact of regional annual average temperature on SES without considering aggregation patterns.

Table S1 Minimal-adequate GLM results of α diversity.

|  | SST | Chl | DO | NH_3_-N | NO_2_-N | NO_3_-N | PO3+ 4 |
| --- | --- | --- | --- | --- | --- | --- | --- |
| 2004 | *** |  | *** |  |  | ** |  |
| 2005 |  |  | *** |  | ** |  |  |
| 2006 |  |  |  |  |  |  | *** |
| 2007 |  |  | ** |  |  |  | * |
| 2008 |  |  | *** |  |  |  | *** |
| 2009 |  |  |  | *** | * |  | *** |
| 2010 |  |  |  |  |  | * |  |
| 2011 |  | ** |  |  | ** | *** |  |
| 2012 |  |  | ** |  |  |  | *** |
| 2013 |  |  |  | ** |  |  | *** |
| 2014 |  |  | *** | ** |  | *** |  |
| 2015 |  |  | *** |  |  | ** | ** |
| 2016 | ** |  |  | ** |  | *** |  |
| 2017 |  |  |  | * |  |  | *** |
| 2018 |  | ** |  | *** |  |  | *** |

Table S2 Formula of β diversity and its partitioning (a: number of shared species; b and c: number of species endemic to each of the two sites, respectively).

| β diversity | Formula |
| --- | --- |
| Sørensen pairwise dissimilarity index (*β_sor_*) | *β_sor_* = (*b* + *c*) / (2*a* + *b* + *c*) |
| Spatial turnover components (*β_sim_*) | *β_sim_* = min (*b*, *c*)/ (a + min (*b*, *c*)) |
| Nestedness components (*β_nes_*) | *β_nes_* = |*b* - *c*|/ (2a + *b* + *c*) × a / (a + min (*b*, *c*)) |
| Similarity (*D*) | *D* = 1- *β_sor_* |

Table S3 Final variables screened by covariance diagnosis and forward selection (- indicating that SST in the year was not statistically significant for diversity. Fifty-first spatial variables were numbered 1 to 51, and the table only shows the serial numbers of the selected spatial variables).

| Year | SST | Nutrient variable | Spatial variable |
| --- | --- | --- | --- |
| 2004 | SST | NH_3_-N | 2~7, 9, 11, 13, 14, 16, 21, 27, 34, 35, |
| 2005 | SST | NH_3_-N, NO_2_-N, NO_3_-N, PO3+ 4 | 1~16, 20, 30, 34, 39, 46, 48 |
| 2006 | - | NO_2_-N, NO_3_-N, PO3+ 4 | 1~8, 10~15, 17, 22, 25 |
| 2007 | - | NH_3_-N, NO_3_-N, PO3+ 4 | 1~8, 10~13, 15, 16, 22, 25, 27, 29, 30, 33~35, 46 |
| 2008 | SST | NO_3_-N, PO3+ 4 | 1~8, 11~13, 16, 17, 22, 23, 29, 31, 33, 46 |
| 2009 | - | NH_3_-N, NO_2_-N, NO_3_-N, PO3+ 4 | 1, 2, 4~10, 12, 13, 15~17 |
| 2010 | - | NO_2_-N, NO_3_-N | 2~8, 11, 15, 17, 19 |
| 2011 | SST | NO_2_-N, NO_3_-N, PO3+ 4 | 1~8, 10~14, 16~19, 22, 24, 25, 29, 33, 35, 46 |
| 2012 | SST | NO_3_-N, PO3+ 4 | 1~10, 12, 14, 16, 17, 21, 24~26, 33, 34, 36, 39 |
| 2013 | SST | NO_3_-N, PO3+ 4 | 1~7, 9, 11, 14, 46 |
| 2014 | - | NH_3_-N, NO_3_-N, PO3+ 4 | 1~8, 12, 14, 17, 23, 24, 31, 33, 41 |
| 2015 | SST | NH_3_-N, NO_3_-N, PO3+ 4 | 1~8, 12, 14, 16, 17, 21, 22, 24, 25, 32~36, 41, 48 |
| 2016 | SST | NH_3_-N, NO_3_-N | 1~9, 12, 17, 48 |
| 2017 | SST | NH_3_-N, PO3+ 4 | 1, 2, 4~7, 12, 14, 17, 41 |
| 2018 | - | NH_3_-N, NO_2_-N, PO3+ 4 | 1~10, 13, 14, 16, 24, 31, 34, 48, 50 |

Table S4 Summary of GAM results for the model of the relationship between the contribution of SST, nutrient variables, spatial variables, all contribution and annual average SST and nutrient levels (All results are for models after generalized cross validation (GCV); Dark blue, light yellow and dark yellow underlines indicate variables that are significant at the 0.1, 0.01 and 0.001 levels respectively.).

| Contribution | Model | SST (k = 4) | | | Nutrient (k = 3) | | | R-sq.(adj) | Deviance  explained | GCV |
| --- | --- | --- | --- | --- | --- | --- | --- | --- | --- | --- |
|  |  | *edf* | *F* value | *P* value | *edf* | *F* value | *P* value |  |  |  |
| SST | SST | 1.000 | 0.71 | 0.414 |  |  |  | -0.042 | 5.35% | 0.0710 |
|  | Nutrient |  |  |  | 1.000 | 0.29 | 0.602 | -0.050 | 2.21% | 0.0734 |
|  | Full model | 1.000 | 0.71 | 0.415 | 1.000 | 0.30 | 0.591 | -0.059 | 7.67% | 0.0813 |
| Nutrient | SST | 1.000 | 0.06 | 0.814 |  |  |  | -0.072 | 0.43% | 0.0815 |
|  | Nutrient |  |  |  | 1.419 | 1.09 | 0.472 | 0.031 | 14.30% | 0.0749 |
|  | Full model | 1.000 | 0.06 | 0.808 | 1.404 | 0.45 | 0.491 | -0.046 | 14.90% | 0.0875 |
| SST+ Nutrient | SST | 1.000 | 0.04 | 0.854 |  |  |  | -0.074 | 0.27% | 0.0853 |
|  | Nutrient |  |  |  | 1.358 | 1.10 | 0.468 | 0.026 | 13.40% | 0.0784 |
|  | Full model | 1.000 | 0.04 | 0.854 | 1.342 | 0.45 | 0.488 | -0.054 | 13.70% | 0.0918 |
| Spatial variables | SST | 1.923 | 0.93 | 0.378 |  |  |  | 0.093 | 26.20% | 0.1673 |
|  | Nutrient |  |  |  | 2.449 | 8.41 | 0.002 | 0.767 | 73.60% | 0.0657 |
|  | Full model | 2.465 | 2.79 | 0.065 | 1.989 | 14.59 | < 0.001 | 0.850 | 86.80% | 0.0479 |
| All | SST | 1.814 | 0.73 | 0.472 |  |  |  | 0.046 | 21.20% | 0.1730 |
|  | Nutrient |  |  |  | 2.387 | 8.72 | 0.002 | 0.769 | 72.90% | 0.6555 |
|  | Full model | 2.010 | 1.39 | 0.239 | 1.987 | 11.25 | 0.002 | 0.805 | 80.90% | 0.6219 |

Table S5 Summary of GAM results for the model of the relationship between the decay effect of SST gradient, nutrient gradients, geographic distance, all and annual average SST and nutrient levels (All results are for models after generalized cross validation (GCV); Dark blue, light blue and light yellow underlines indicate variables that are significant at the 0.1, 0.05 and 0.01 levels respectively. The green underline is the model shown in the main figure in the text.).

| Decay effect | Model | SST (k = 4) | | | Nutrient (k = 3) | | | R-sq.(adj) | Deviance explained | GCV |
| --- | --- | --- | --- | --- | --- | --- | --- | --- | --- | --- |
|  |  | *edf* | *F* value | *P* value | *edf* | *F* value | *P* value |  |  |  |
| SST gradient | SST | 2.832 | 3.28 | 0.064 |  |  |  | 0.600 | 52.10% | 0.0314 |
|  | Nutrient |  |  |  | 1.000 | 0.48 | 0.502 | -0.028 | 5.22% | 0.0458 |
|  | Full model | 2.941 | 6.71 | 0.009 | 1.000 | 6.70 | 0.027 | 0.787 | 74.50% | 0.0209 |
| Nutrient gradients | SST | 2.758 | 2.51 | 0.113 |  |  |  | 0.394 | 43.30% | 0.0478 |
|  | Nutrient |  |  |  | 1.000 | 1.20 | 0.293 | 0.042 | 12.20% | 0.0554 |
|  | Full model | 2.855 | 5.32 | 0.020 | 1.040 | 5.50 | 0.044 | 0.631 | 62.80% | 0.0388 |
| SST + nutrient gradients | SST | 2.725 | 1.87 | 0.195 |  |  |  | 0.325 | 38.30% | 0.0612 |
|  | Nutrient |  |  |  | 1.000 | 1.85 | 0.197 | 0.106 | 17.30% | 0.0618 |
|  | Full model | 2.885 | 6.15 | 0.012 | 1.000 | 10.03 | 0.010 | 0.660 | 65.70% | 0.0423 |
| Geographic distance | SST | 1.000 | 0.15 | 0.708 |  |  |  | -0.067 | 1.25% | 0.0563 |
|  | Nutrient |  |  |  | 1.000 | 7.11 | 0.019 | 0.368 | 38.00% | 0.0353 |
|  | Full model | 1.000 | 0.21 | 0.654 | 1.000 | 6.48 | 0.026 | 0.352 | 39.30% | 0.0406 |
| All | SST | 1.000 | 0.02 | 0.883 |  |  |  | -0.075 | 0.19% | 0.0639 |
|  | Nutrient |  |  |  | 1.000 | 6.35 | 0.026 | 0.346 | 36.10% | 0.0409 |
|  | Full model | 1.000 | 0.03 | 0.872 | 1.000 | 5.80 | 0.033 | 0.300 | 36.20% | 0.0479 |

Table S6 Summary of GAM results for the model of the relationship between the standardized effect size (SES) and SST (All results are for models after generalized cross validation (GCV); Light blue underlines indicate variables that are significant at the 0.05 levels. The green underline is the model shown in the main figure in the text.).

| Model | SST (k = 4) | | | Nutrient (k = 3) | | | R-sq.(adj) | Deviance explained | GCV |
| --- | --- | --- | --- | --- | --- | --- | --- | --- | --- |
|  | *edf* | *F* value | *P* value | *edf* | *F* value | *P* value |  |  |  |
| SST | 1.036 | 5.59 | 0.034 |  |  |  | 0.258 | 31.30% | 2.0851 |
| Nutrient |  |  |  | 1.000 | 0.12 | 0.733 | -0.067 | 0.93% | 2.9909 |
| Full model | 1.137 | 9.61 | 0.012 | 3.107 | 2.35 | 0.160 | 0.509 | 65.80% | 1.3300 |


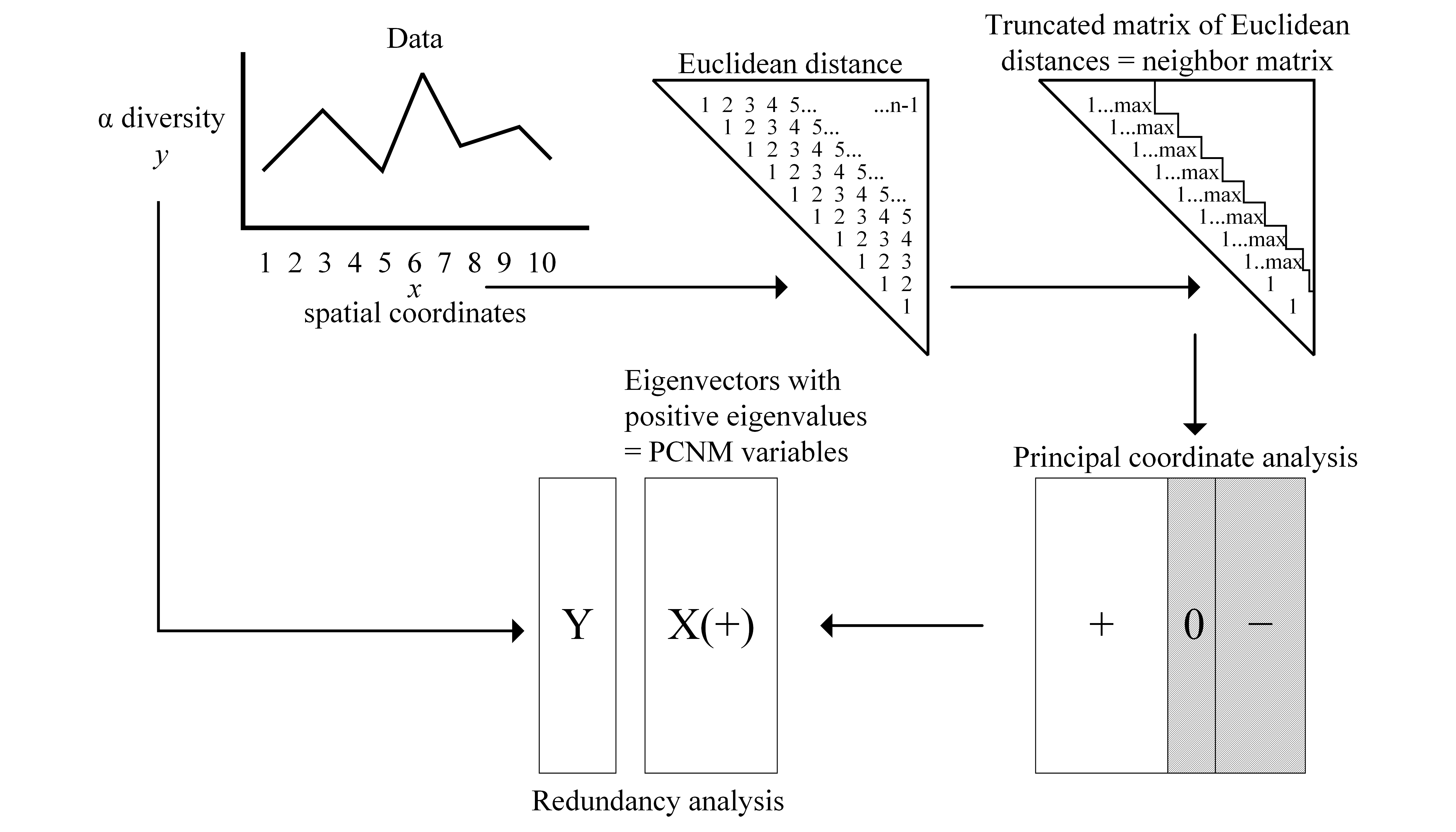


Fig. S1 Calculation of PCNM variables (modified from Borcard et al. 2004).


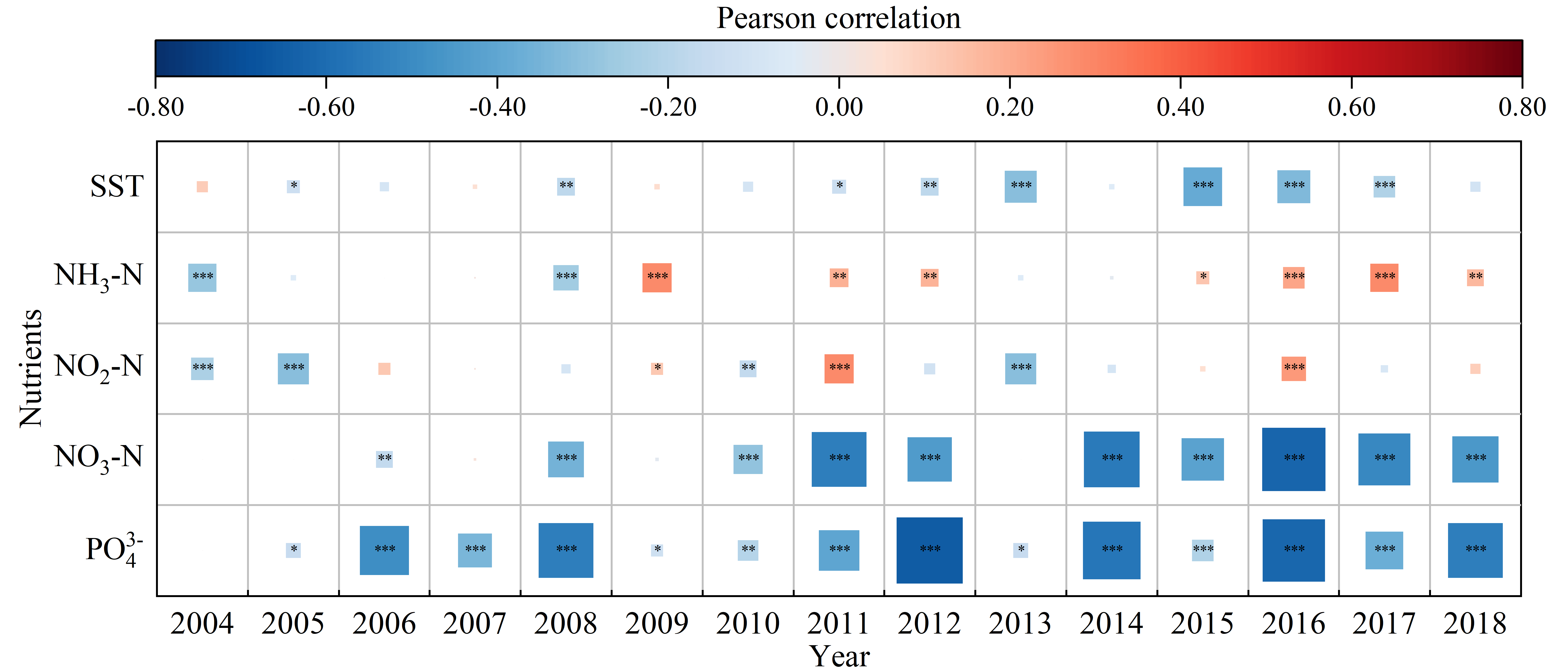


Fig. S2 The Pearson correlation between nutrients and Shannon-Wiener diversity every year (*, ** and *** denotes the significant correlation at the 0.05, 0.01 and 0.001 level.)


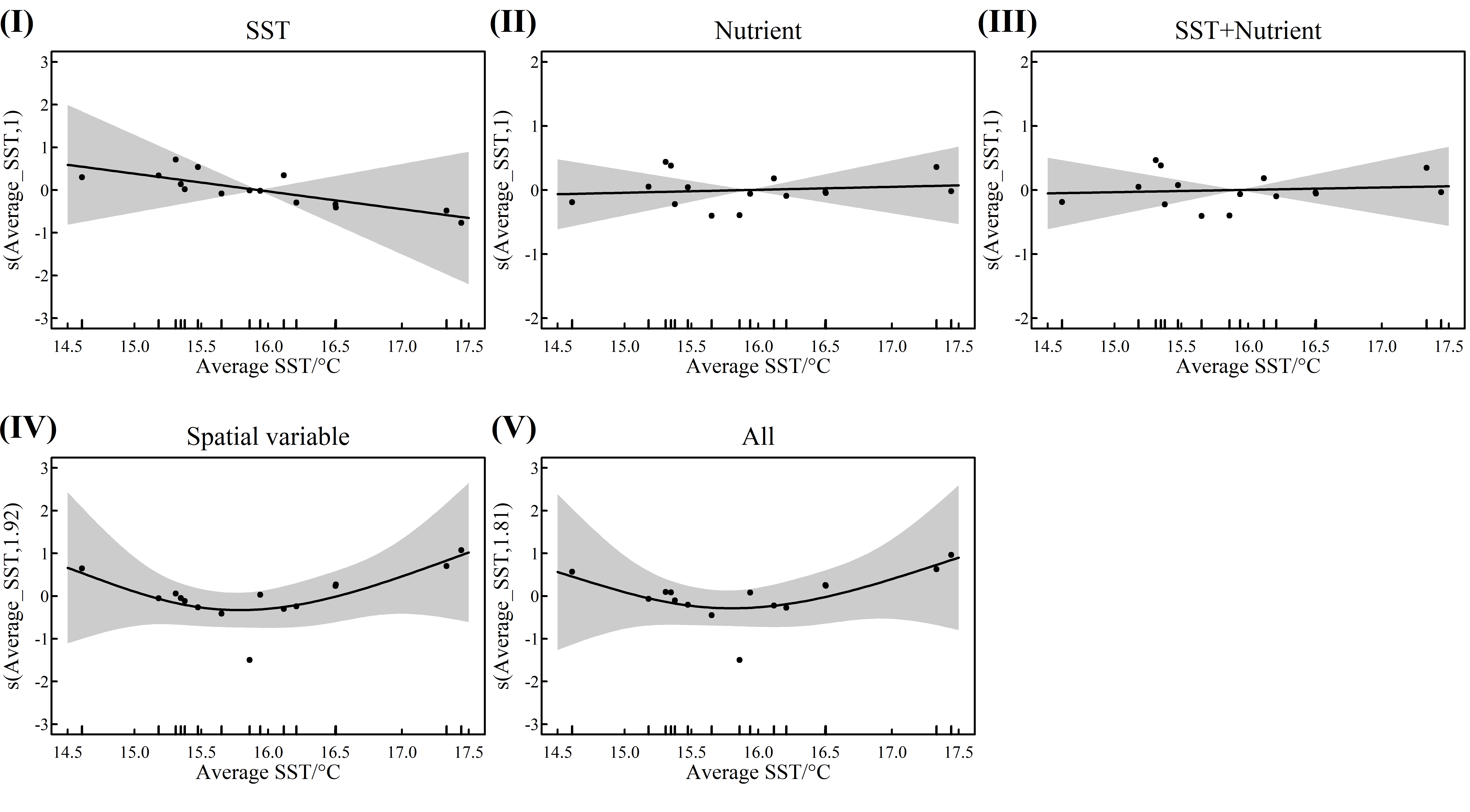


Fig. S3 The effect of regional annual average SST on the relative contribution of processes in the construction of α diversity, using univariate GAMs analysis.


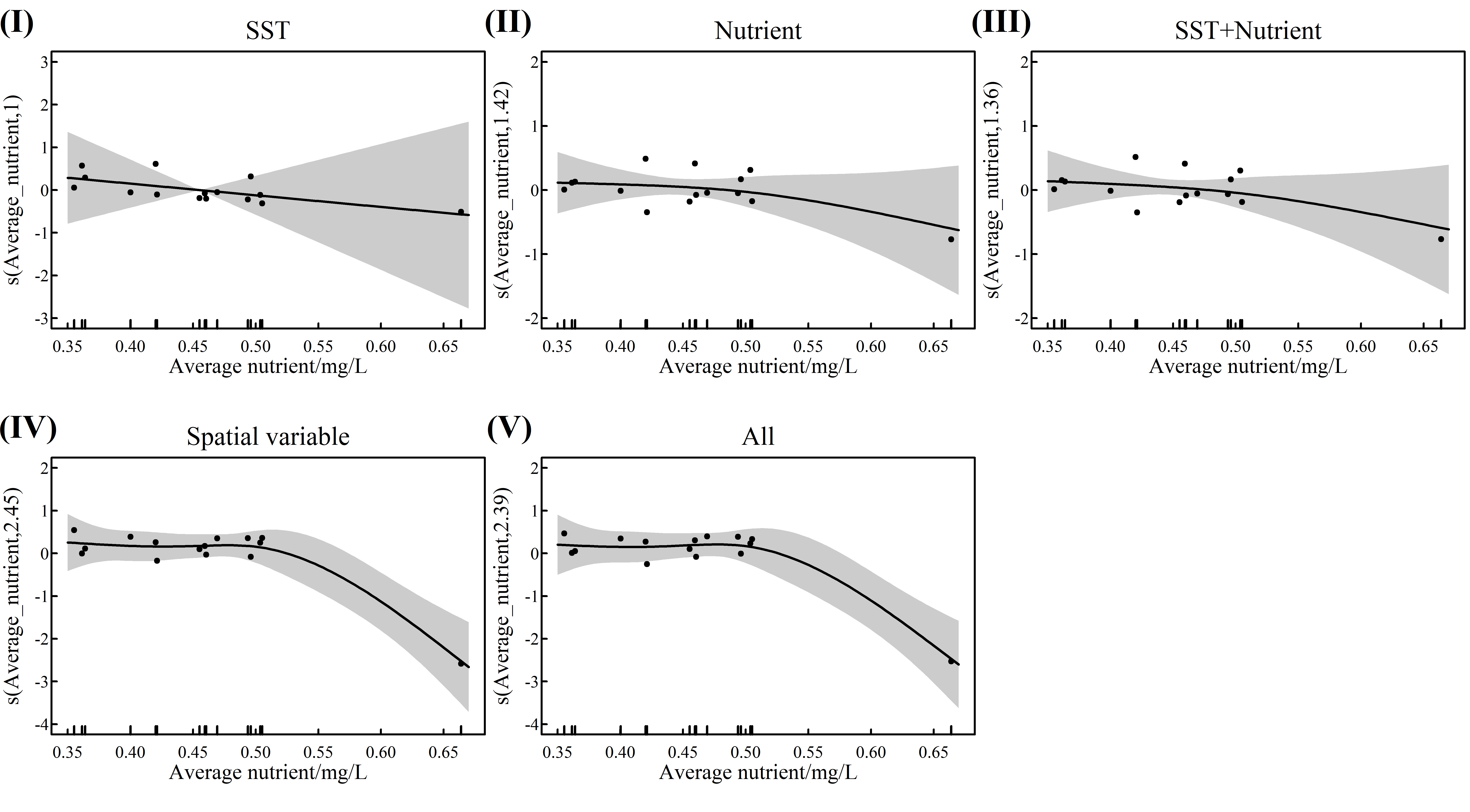


Fig. S4 The effect of regional annual average nutrient levels on the relative contribution of processes in the construction of α diversity, using univariate GAMs analysis.


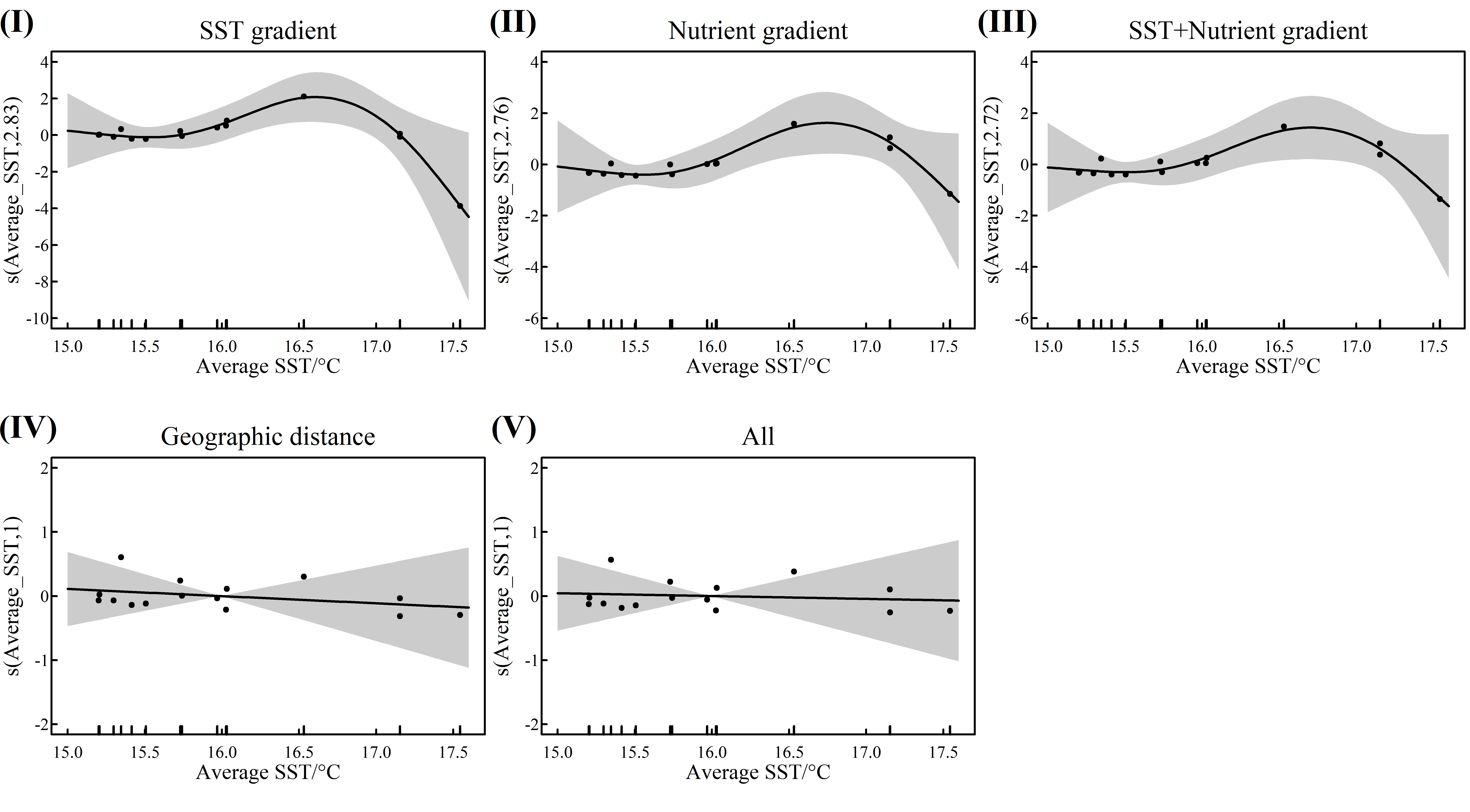


Fig. S5 The effect of regional annual average SST on the relative decay effects of β diversity, using univariate GAMs analysis.


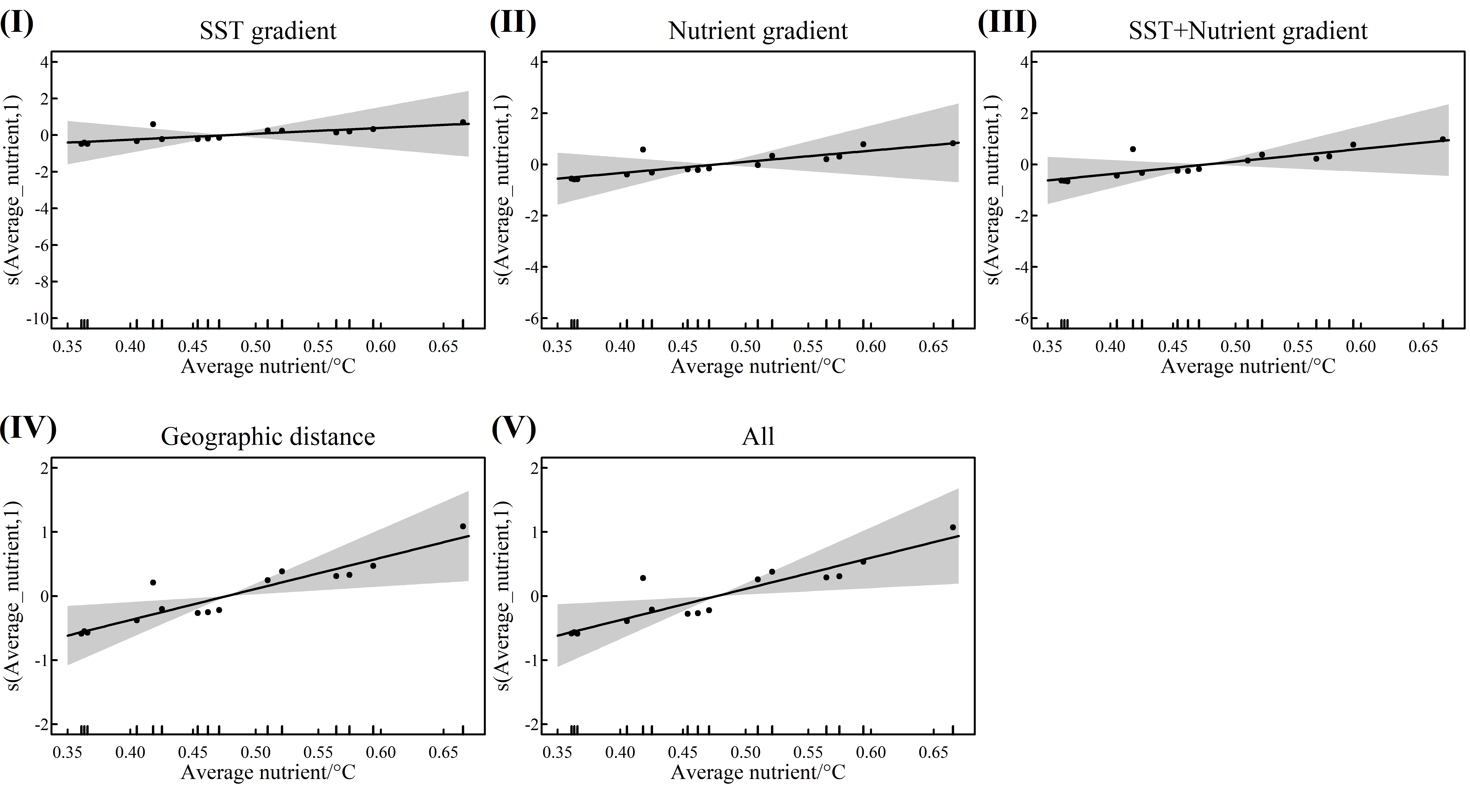


Fig. S6 The effect of regional annual average nutrient levels on the relative decay effects of β diversity, using univariate GAMs analysis.


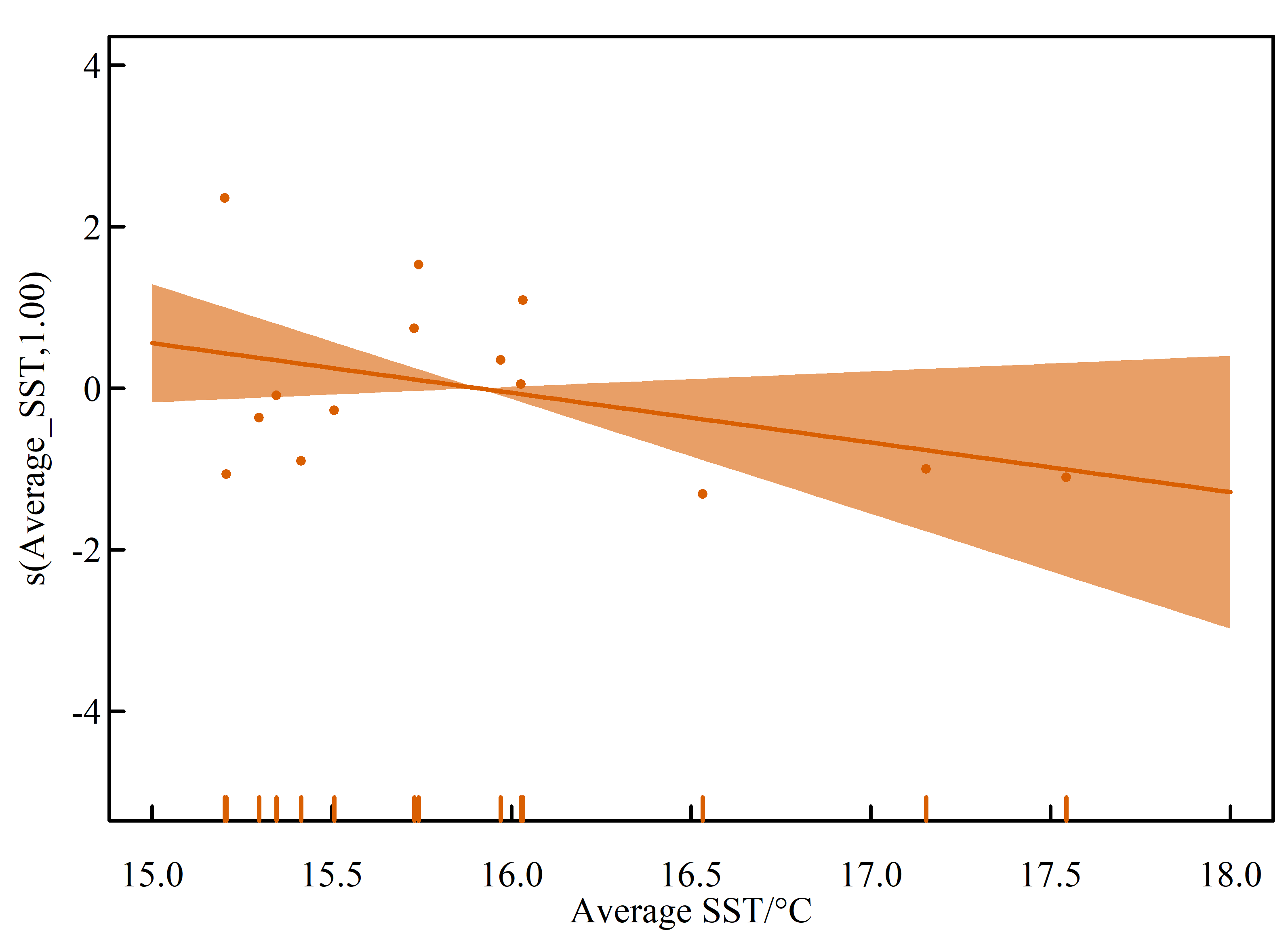


Fig. S7 Impact of regional annual average temperature on SES without considering aggregation patterns.
